# Profiling of Childhood Adversity-Associated DNA Methylation Changes in Alcoholic Patients and Healthy Controls

**DOI:** 10.1371/journal.pone.0065648

**Published:** 2013-06-14

**Authors:** Huiping Zhang, Fan Wang, Henry R. Kranzler, Hongyu Zhao, Joel Gelernter

**Affiliations:** 1 Department of Psychiatry, Yale University School of Medicine, New Haven, Connecticut, United States of America; 2 Department of Genetics, Yale University School of Medicine, New Haven, Connecticut, United States of America; 3 Department of Neurobiology, Yale University School of Medicine, New Haven, Connecticut, United States of America; 4 Division of Biostatistics, Yale University School of Public Health, New Haven, Connecticut, United States of America; 5 VA Connecticut Healthcare System, West Haven, Connecticut, United States of America; 6 Department of Psychiatry, University of Pennsylvania Perelman School of Medicine and VISN4 MIRECC, Philadelphia VAMC, Philadelphia, Pennsylvania, United States of America; Dartmouth Medical School, United States of America

## Abstract

The increased vulnerability to alcohol dependence (AD) seen in individuals with childhood adversity (CA) may result in part from CA-induced epigenetic changes. To examine CA-associated DNA methylation changes in AD patients, we examined peripheral blood DNA methylation levels of 384 CpGs in promoter regions of 82 candidate genes in 279 African Americans [AAs; 88 with CA (70.5% with AD) and 191 without CA (38.2% with AD)] and 239 European Americans [EAs; 61 with CA (86.9% with AD) and 178 without CA (46.6% with AD)] using Illumina GoldenGate Methylation Array assays. The effect of CA on methylation of individual CpGs and overall methylation in promoter regions of genes was evaluated using a linear regression analysis (with consideration of sex, age, and ancestry proportion of subjects) and a principal components-based analysis, respectively. In EAs, hypermethylation of 10 CpGs in seven genes (*ALDH1A1*, *CART*, *CHRNA5*, *HTR1B*, *OPRL1*, *PENK*, and *RGS19*) were cross validated in AD patients and healthy controls who were exposed to CA. *P* values of two CpGs survived Bonferroni correction when all EA samples were analyzed together to increase statistical power [*CHRNA5_*cg17108064: *P_adjust_* = 2.54×10^−5^; *HTR1B*_cg06031989: *P_adjust_* = 8.98×10^−5^]. Moreover, overall methylation levels in the promoter regions of three genes (*ALDH1A1*, *OPRL1* and *RGS19*) were elevated in both EA case and control subjects who were exposed to CA. However, in AAs, CA-associated DNA methylation changes in AD patients were not validated in healthy controls. Our findings suggest that CA could induce population-specific methylation alterations in the promoter regions of specific genes, thus leading to changes in gene transcription and an increased risk for AD and other disorders.

## Introduction

Childhood adversity (CA) may lead to impaired mental and physical health that can persist into adulthood. Adverse childhood experiences have been associated with mood disorders [1], schizophrenia [2], [3], [4], anxiety disorders [5], suicide [6], personality disorders [7], [8], posttraumatic stress disorder [9], [10], and substance use disorders [11], [12], [13], [14]. Excessive and persistent adversity in early childhood produces sustained elevations of stress hormones (e.g., cortisol) [15], which may damage the development of the basic neural circuitry in the brain.

The effect of CA on vulnerability to the varieties of disorders may be mediated in part by epigenetic events, such as DNA methylation, histone modification and microRNA regulation, which can substantially affect gene transcription without changing DNA sequence [16], [17]. Epigenetic modifications are essential for cellular development and differentiation in mammals [18], [19]. However, epigenetic changes resulting from environmental factors such as CA may alter biological activities in the brain, leading to increased risk for diseases. A seminal study by Weaver et al. [20] showed that early life stress (i.e., postnatal maternal separation) in rats led to higher methylation levels at specific CpG sites in the exon 1_7_ promoter of the glucocorticoid receptor (GR) gene (*GR*), decreased *GR* expression in the hippocampus, and increased hypothalamic-pituitary-adrenal (HPA) responses to stress. A follow-up study demonstrated that DNA methylation was altered in a broad chromosomal area (seven million base pairs) containing the GR gene in rats receiving poor maternal care [21]. Thus, subjects exposed to CA may have a lowered threshold for activation of the stress response system and experience stress more readily than those unexposed to CA.

Epigenetic changes in the *GR* gene have also been observed in humans who experienced CA. Greater methylation of specific CpGs in the exon 1_F_ promoter of the human *GR* gene (*NR3C1*) and lower expression levels of *NR3C1* were observed in postmortem hippocampus of suicide completers with a history of childhood abuse compared to either suicide completers with no childhood abuse or non-suicidal controls [22]. Moreover, altered methylation of specific CpGs in the exon 1_F_ promoter of *NR3C1* also appeared in peripheral blood leukocytes of healthy adults who experienced stress in early childhood [23]. These findings suggest that CA may exert a common effect on epigenetic regulation of *GR* expression in both brain and peripheral tissues. Thus, the use of noninvasive measurements of peripheral blood samples could provide a highly feasible method to examine CA-associated epigenetic changes of certain genes.

CA may also lead to differential methylation of genes involved in other biological pathways. McGowan et al. [24] found hypermethylation and reduced expression of the ribosomal RNA gene (*rRNA*) in postmortem hippocampus of suicide completers who had experienced early childhood neglect or abuse. Beach et al. [25] reported a significant association between childhood abuse and hypermethylation of the CpG island upstream of the serotonin transporter gene. Additionally, a genome-wide methylation analysis of a small sample (n = 50) using methylated DNA immuneprecipitation (MeDIP) demonstrated that numerous genes involved in key cell signaling pathways were differentially methylated in individuals who were reared in disadvantaged socio-economic conditions [26].

The existing literature is limited in that either only a single gene was examined or the sample size was small in these studies. An additional and pressing concern is that CA-associated methylation changes may be confounded by comorbid disorders. For example, there is evidence that CA is associated with abuse of or dependence on multiple substances [11], [12], [13], [14]. We recently observed DNA methylation alterations in specific genes in patients with alcohol dependence (AD) by examining methylation levels of 384 CpGs in 82 candidate genes using the Illumina GoldenGate methylation array [27]. However, given a high rate of CA (more than 70%) in these AD patients, the alterations in DNA methylation that we observed may have been due to CA, AD, or both. The purpose of the present study was to identify CpG sites whose methylation changes were attributable specifically to CA. To achieve this, we compared methylation levels of these 384 CpG sites in 82 candidate genes between AD patients with and without CA as well as between healthy controls with and without CA, respectively. Specific CpG sites (or genes) with altered methylation in both AD patients and healthy controls due to exposure to CA were identified.

## Materials and Methods

### Ethics Statement

The study protocol was approved by each local institutional review board (the Institutional Review Board of the University of Connecticut, the Yale University Human Research Protection Program, and the Institutional Review Board for Human Research of the Medical University of South Carolina), and written informed consent was obtained from each subject.

### Subject Recruitment

Two hundred seventy-nine African Americans (AAs) and 239 European Americans (EAs) were recruited from substance abuse treatment centers and through advertisements at the University of Connecticut Health Center (n = 257), the Medical University of South Carolina (n = 134) and Yale University School of Medicine (n = 127) (**Table 1**). As described by Xie et al. [14], childhood adversity (CA) was assessed by selected certain questions (Z1B, Z3, Z4A and Z4B) from Section Z (Z1–Z11) of the Semi-structured Assessment for Drug Dependence and Alcoholism (SSADDA) [28] to investigate the social environment when subjects were growing up. Basically, CA assessment consisted of four questions related to more severe adversity experiences: 1) Z1B: Did either of your parents die before you were age 6; 2) Z3: Did you ever witness or experience a violent crime, like a shooting or a rape, by age 13; 3) Z4A: By the time you were age 13, were you ever sexually abused; and 4) Z4B: By the time you were age 13, were you ever beaten by an adult so badly that you needed medical care, or had marks on your body that lasted for more than 30 days. Individuals who endorsed any of these adverse childhood experiences were coded as positive for exposure to CA. Of the 279 AAs, 88, or nearly one-third were exposed to CA; of the 239 EAs, 61, or about one-quarter, were exposed to CA. Information from the SSADDA was also used to derive a lifetime diagnosis of alcohol dependence (AD) according to the criteria of the Diagnostic and Statistical Manual of Mental Disorders, 4th edition (DSM-IV) [29]. One hundred thirty-five AAs (48.4%) and 136 EAs (56.9%) were affected with AD. Information on comorbid substance (cocaine, opioid, marijuana, or nicotine) dependence is also presented in **Table 1**. All subjects were not affected with a lifetime major psychotic disorder (schizophrenia or bipolar disorder). Most subjects (271 AD cases and 144 healthy control) included in this study were also included in our previous study [27], which focused on studying AD-associated DNA methylation alterations, and 103 healthy controls were new.

**Table 1:** Characteristics of African American (AA) and European American (EA) samples.

	AA cases with AD		AA controls		EA cases with AD		EA controls	
	With CA	Without CA	With CA	Without CA	With CA	Without CA	With CA	Without CA
	(n = 62)	(n = 73)	(n = 26)	(n = 118)	(n = 53)	(n = 83)	(n = 8)	(n = 95)
Parent death, n (%)	7 (11.3)^a^	0 (0)	6 (23.1)	0 (0)	8 (15.1)	0 (0)	2 (25.0)	0 (0)
Witness Violence, n (%)	44 (71.0)^a^	0 (0)	9 (34.6)	0 (0)	20 (37.7)	0 (0)	2 (25.0)	0 (0)
Sex or physical abuse, n (%)	30 (48.3)^a^	0 (0)	14 (53.8)	0 (0)	43 (81.1)	0 (0)	5 (62.5)	0 (0)
With 1 adversity, n (%)	42 (67.7)^b^	0 (0)	23 (88.5)	0 (0)	30 (56.6)	0 (0)	7 (87.5)	0 (0)
With 2 adversities, n (%)	17 (27.4)^b^	0 (0)	3 (11.5)	0 (0)	17 (32.1)	0 (0)	1 (12.5)	0 (0)
With 3 adversities, n (%)	3 (4.8)^b^	0 (0)	0 (0)	0 (0)	6 (11.3)	0 (0)	0 (0)	0 (0)
Comorbid CD, n (%)	55 (88.7)^c^	71 (97.3)^d^	0 (0)	0 (0)	27 (50.9)	38 (45.8)	0 (0)	0 (0)
Comorbid OD, n (%)	10 (16.1)^c^	6 (8.2)^d^	0 (0)	0 (0)	11 (20.8)	16 (19.3)	0 (0)	0 (0)
Comorbid MjD, n (%)	27 (43.5)^c^	20 (27.4 )^d^	0 (0)	0 (0)	11 (20.8)	28 (33.7)	0 (0)	1 (1.1)
Comorbid ND, n (%)	54 (87.1)^c^	58 (79.5)^d^	0 (0)	5 (4.2)	39 (73.6)	57 (68.7)	1 (12.5)	11 (11.6)
Sex, male, n (%)	35 (56.5)	34 (46.6)	3 (11.5)	29 (24.6)	27 (50.9)	46 (55.4)	3 (37.5)	50 (52.6)
Age, years	43±9	42±7	39±15	36±13	43±11	41±13	33±15	37±16

CA: childhood adversity [i.e., (1) parents died before age 6, (2) witnessed or experienced a violent crime (like a shooting or a rape) by age 13, or (3) sexually or badly physically abused by age 13].

AD: alcohol dependence; CD: cocaine dependence; OD: opioid dependence; MjD: marijuana dependence; ND: nicotine dependence.

a The distribution of adversity types in AA cases (with CA) was significantly different from that in EA cases (with CA) by Chi-square test (*P*≤0.05).

b The distribution of numbers of adversities in AA cases (with CA) was significantly different from that in EA cases (with CA) by Chi-square test (*P*≤0.05).

c The distribution of comorbid substance dependence (CD, OD, MjD, or ND) in AA cases (with CA) was significantly different from that in EA cases (with CA) by Chi-square test (*P*≤0.05).

d The distribution of comorbid substance dependence (CD, OD, MjD, or ND) in AA cases (without CA) was significantly different from that in EA cases (without CA) by Chi-square test (*P*≤0.05).

To verify the self-reported race of subjects, we used a Bayesian model-based clustering method implemented in the program STRUCTURE [30] to estimate the African and European ancestry proportions of individual subjects, using genotype data from 41 ancestry informative markers (AIMs), including 36 short tandem repeat markers and five single nucleotide polymorphisms (SNPs), as described previously [9], [31]. If their African ancestry proportion scores were ≥0.5, subjects were classified as AAs; if their African ancestry proportion scores were <0.5, subjects were classified as EAs. We excluded subjects if their ancestry proportion scores did not match their self-reported races. To minimize the influence of genetic background of subjects on DNA methylation analysis results, ancestry proportions of subjects were considered as a covariate in the linear regression model (see below).

### Genomic DNA Extraction and Bisulfite Modification

Genomic DNA was extracted from peripheral blood using the PAXgene Blood DNA Kit (PreAnalytiX, Hombrechtikon, Switzerland). One µg of genomic DNA was treated with the bisulfite reagent included in the EZ DNA Methylation Kit (Zymo Research, Orange, CA, USA). Unmethylated cytosines were converted to uracils, while methylated cytosines remained unchanged [32]. Bisulfite-converted DNA samples were then used in the Illumina GoldenGate Methylation assay.

### Illumina GoldenGate Methylation Assay

As described in our recent publication [27], the Illumina GoldenGate methylation assay was used to analyze methylation levels of 384 CpG sites located in promoter regions [from 2,000 bp upstream to 1,000 bp downstream of the transcription start sites (TSS)] of 82 candidate genes. Briefly, 1 µg of bisulfite-converted genomic DNA from each subject was spotted on the methylation chip (32 samples per chip). The experiment was conducted at the Yale Center for Genome Analysis (YCGA) (West Haven Campus, Yale University), following the standard Illumina protocol. These genes are involved in several major brain neurotransmission systems (e.g., the dopaminergic, opioidergic, serotonergic, GABAergic/glutamatergic, cholinergic, and cannabinoid systems), alcohol metabolism, DNA methylation, or signal transduction. On average, about five CpGs per gene were included in this custom methylation profiling panel. The array-based methylation assay was conducted using the standard Illumina protocol. Information of these 384 CpG sites is listed in **Table S1**.

Image processing and intensity data extraction were performed using the Illumina GenomeStudio™ Methylation Module v.1.0 Software. The background normalization algorithm was used to minimize background variation within the array by using built-in negative control signals. The methylation level (defined as β = [Max(Cy5,0)]/[Max(Cy3,0)+Max(Cy5,0)+100]) of each individual CpG site was estimated as the ratio of intensities between methylated and unmethylated alleles. It ranges from 0 in the case of completely unmethylated sites to 1 in completely methylated sites. To monitor both bisulfite conversion efficiency and accuracy of methylation detection, internal and technical controls were included in the methylation assay. The internal controls consisted of methylated and non-methylated human DNA standards (Zymo Research, Orange, CA, USA). 5% of the bisulfite-converted human DNA samples (518×5% = 26) were replicated in the DNA methylation assay, and these were considered as technical controls. CpG methylation assays were highly reproducible within arrays (r^2^ = 0.995) and between arrays (r^2^ = 0.992). Additionally, methylation-array-quantified methylation levels of one gene [the serotonin receptor 3A gene (*HTR3A*)] were validated using the Sequenom MassARRAY EpiTYPER approach (Sequenom, San Diego, CA, USA) [27].

### Genotype-CA Interaction on DNA Methylation

To examine whether CA-associated DNA methylation changes could be modulated by genetic factors, a functional single nucleotide polymorphism in the exon 5 of the nicotinic acetylcholine receptor subunit alpha-5 gene (*CHRNA5* rs16969968) [33] was genotyped in all samples by the TaqMan method [34]. The interactive effect of genotypes of SNP rs16969968 and CA on methylation levels of *CHRNA5* promoter *CpG* cg17108064 (one of the top CpGs whose methylation levels were significantly influenced by CA, as described below in the Results section) was further analyzed.

### DNA Methylation Data Analysis

All statistical analyses were implemented using the open-source program R 2.15.2 (http://www.r-project.org/). As described in published studies [35], [36], DNA methylation raw data were first processed by applying function “ComBat” in the R package sva [37] to control for known batch effects (potentially observed across multiple batches of microarray experiments), in which CA was considered as the phenotype of interest and the output was a similar matrix to that of the input, where batch effects were removed. An empirical Bayes-moderated t approach, implemented in the Bioconductor package Limma [38], was used to analyze differential methylation of individual CpG sites as a function of CA. To assess whether the observed DNA methylation differences were influenced by confounding factors, the adjusted *P* value (*P*_adjust_) was calculated for each CpG by multiple linear regression analysis, in which CA and confounding factors (sex, age, AD status, and ancestry proportion) were considered as explanatory variables. To confirm that DNA methylation alterations were specifically induced by CA, we first examined CA-associated DNA methylation changes in AD cases and then replicated the findings in healthy controls. Correction for multiple testing was performed using the QVALUE software [39]. The *P* values obtained from the above analyses were evaluated and presented as *q* values by controlling the false discovery rate at 0.05. Given the evidence that DNA methylation is race specific [40], [41], [42], the above analyses were performed separately in AAs and EAs to adjust for influence of race on association between DNA methylation and CA.

Additionally, a principal components-based analysis was carried out to examine CA-associated overall methylation changes in each gene by integrating the methylation status of individual CpG sites in their promoter regions [43]. First, CpG sites were assigned to their genes based on annotation files of the Illumina GoldenGate Methylation assay. Second, a principal components-based analysis [44] was applied to each gene using methylation data from all mapped CpGs in that gene. The first principal component (PC1) was used to represent the overall methylation level of the promoter region of the assayed gene. Finally, PC1 was considered as a variable and used to compare overall methylation differences of genes as a function of CA.

## Results

### CA-associated CpG Methylation Changes in AD Cases

**Figure 1:**
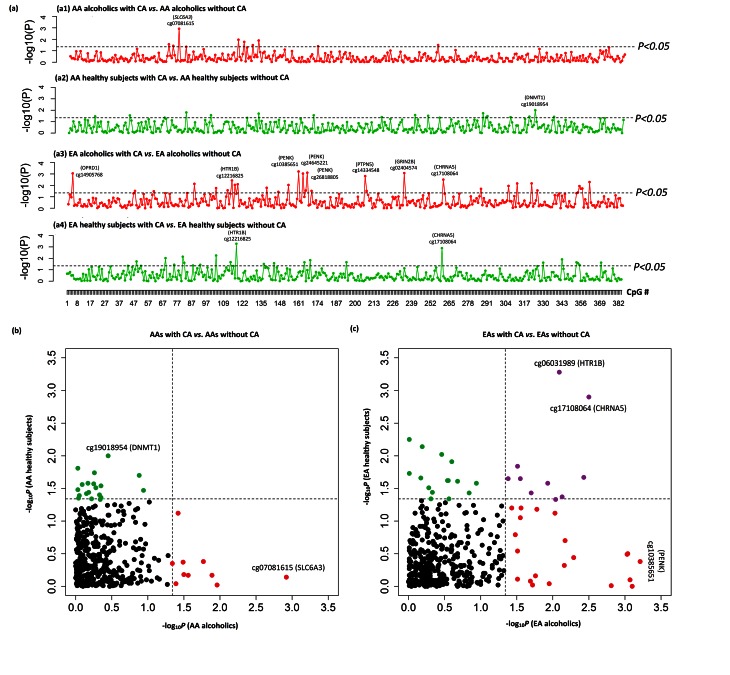
Childhood adversity (CA)-associated methylation alterations in 384 CpGs in promoter regions of 82 candidate genes. a1–a4) Subgroup analysis of CA-associated methylation changes in 384 CpGs in AA alcoholics (a1), AA healthy subjects (a2), EA alcoholics (a3), and EA healthy subjects (a4). CpGs with *P* values <0.005 are shown. X axis: the ID of 384 CpGs; Y axis: −log_10_ (*P* value) (adjusted *P* values obtained from linear regression analysis). Dashed lines indicate that *P* = 0.05. b and c) Scatter plot of –log_10_ (*P* value) of 384 CpGs in AAs (b) and EAs (c). X axis: −log_10_ (*P* value) of 384 CpGs in AA or EA alcoholics; Y axis: –log_10_ (*P* value) of 384 CpGs in AA or EA healthy subjects.

In AA cases with AD, the results of empirical Bayes-moderated t approach indicated that 13 CpGs in 11 genes were differentially methylated due to exposure to CA (*P*_nominal_<0.05). After adjustment for sex, age, and ancestry proportion of subjects using linear regression analysis, *P* values of 10 CpGs in eight genes remained significant (*P*_adjust_<0.05) (**Figure 1a, 1b** and **Table S2)**. In EA cases with AD, the results of empirical Bayes-moderated t approach indicated that 46 CpGs in 31 genes were differentially methylated due to exposure to CA (*P*_nominal_<0.05). After adjustment for sex, age, and ancestry proportion of subjects using linear regression analysis, *P* values of 34 CpGs in 24 genes remained significant (*P*_adjust_<0.05) (**Figure 1a, 1c** and **Table S3)**. However, the *P* values of CpG sites did not survive multiple testing corrections in either population (q>0.05).

### CA-associated CpG Methylation Changes in Healthy Controls

In AA healthy controls, by the empirical Bayes-moderated t approach, 13 CpGs in 13 genes showed differential methylation due to exposure to CA (*P*_nominal_<0.05). When linear regression analysis was performed with the consideration of sex, age, and ancestry proportion of subjects, *P* values of 19 CpGs in 18 genes turned to be significant (*P*_adjust_<0.05) (**Figure 1a, 1b** and **Table S4)**. In EA healthy controls, by the empirical Bayes-moderated t approach, 31 CpG sites in 23 genes showed differential methylation due to exposure to CA (*P*_nominal_<0.05). When linear regression analysis was performed with the consideration of sex, age, and ancestry proportion of subjects, *P* values of 26 CpGs in 21 genes turned to be significant (*P*_adjust_<0.05) (**Figure 1a, 1c** and **Table S5)**. However, the *P* values of CpG sites did not survive multiple testing corrections in either population (q>0.05).

### CA-associated CpG Methylation Changes in both AD Cases and Healthy Controls

In AAs, CA-associated CpG methylation changes in AD cases were not validated in healthy controls (**Figure 1b**). In EAs, 10 CpG sites in seven genes (*ALDH1A1*, *CART*, *CHRNA5*, *HTR1B*, *OPRL1*, *PENK*, and *RGS19*) were differentially methylated in both AD case and control subjects who were exposed to CA (**Figure 1c** **and** **Table 2**). *P* values of two CpGs in two genes survived Bonferroni correction when both EA case and control samples were analyzed together [cg17108064 (*CHRNA5*): *P*_adjust_ = 2.54×10^−5^ and cg06031989 (*HTR1B*): *P*_adjust_ = 8.98×10^−5^]. Higher methylation levels of these 10 CpGs were seen in both EA case and control subjects who were exposed CA (**Figure S1**).

**Table 2:** Differentially methylated CpGs in European American (EA) alcoholics and healthy controls who were exposed to childhood adversity (CA).

CpG site	Genes	EA alcoholics (*N* = 136)			EA healthy controls (*N* = 103)			All EA subjects (*N* = 239)		
		β +CA^a^	β −CA^b^	*P_adj_*	β +CA^a^	β −CA^b^	*P_adj_*	β +CA^a^	β −CA^b^	*P_adj_* c
cg17108064	*CHRNA5*	0.063	0.053	0.003	0.068	0.047	0.001	0.064	0.05	0.00002^d^
cg06031989	*HTR1B*	0.047	0.040	0.008	0.056	0.04	0.0005	0.048	0.04	0.00009^d^
cg12216825	*HTR1B*	0.015	0.012	0.004	0.015	0.011	0.021	0.015	0.011	0.0002
cg08354950	*CART*	0.024	0.019	0.007	0.025	0.019	0.042	0.024	0.019	0.0005
cg00314411	*OPRL1*	0.137	0.121	0.012	0.158	0.126	0.026	0.139	0.123	0.0009
cg12215457	*HTR1B*	0.09	0.084	0.009	0.094	0.082	0.046	0.091	0.083	0.001
cg12902246	*RGS19*	0.185	0.172	0.020	0.197	0.171	0.037	0.187	0.171	0.002
cg16206611	*ALDH1A1*	0.079	0.072	0.031	0.094	0.071	0.015	0.081	0.071	0.002
cg24377504	*OPRL1*	0.038	0.033	0.042	0.04	0.031	0.022	0.038	0.032	0.004
cg08754521	*PENK*	0.023	0.017	0.028	0.026	0.017	0.022	0.023	0.017	0.004

a Methylation levels (β) of CpGs in subjects who were exposed to childhood adversity (+CA).

b Methylation levels (β) of CpGs in subjects who were not exposed to childhood adversity (−CA).

c *P_adj_* was the adjusted *P* value calculated using multivariate linear regression analysis with adjustment of sex, age, ancestry proportion.

d *P_adj_* survived Bonferroni corrections.

### CA-associated Overall Methylation Changes in Gene Promoter Regions

**Figure 2:**
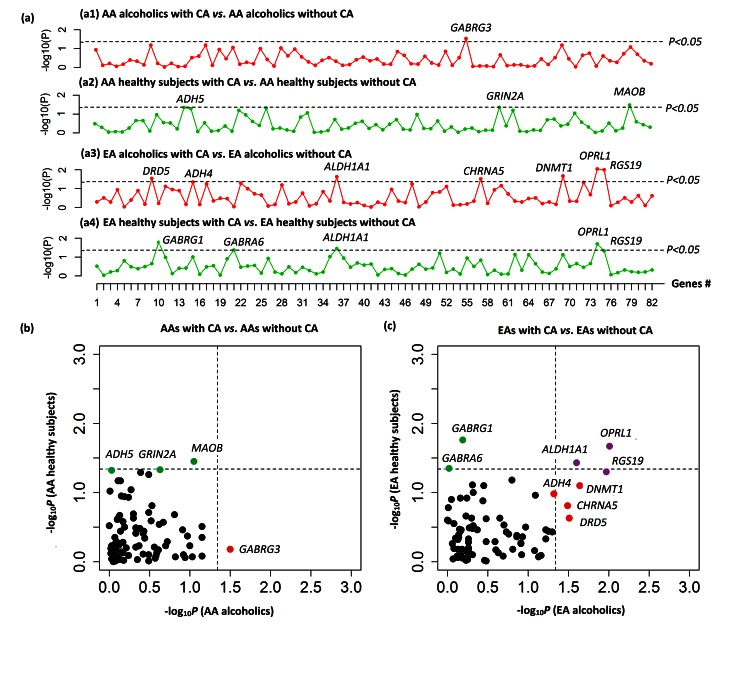
Childhood adversity (CA)-associated overall methylation alterations in promoter regions of 82 candidate genes. (a1–a4) Subgroup analysis of CA-associated overall methylation changes in promoter regions of 82 genes in AA alcoholics (a1), AA healthy subjects (a2), EA alcoholics (a3), and EA healthy subjects (a4). Genes with *P* values <0.05 are listed. X axis: the ID of genes; Y axis: −log_10_ (*P* value) (adjusted *P* values obtained from linear regression analysis). Dashed lines indicate that *P* = 0.05. b and c) Scatter plot of −log_10_ (*P* value) of 82 genes in AAs (b) and EAs (c). X axis: −log_10_ (*P* value) of 82 genes in AA or EA alcoholics; Y axis: −log_10_ (*P* value) of 82 genes in AA or EA healthy subjects.

In AA cases with AD, only the promoter region of *GABRG3* showed hypermethylation in association with CA (*P*_adjust = _0.032) (**Figure 2a** **and Table S6**). In AA control subjects, promoter regions of three genes (*ADH5, GRIN2A* and *MAOB*) were hypermethylated in the presence of exposure to CA (*P*_adjust = _0.024–0.047) (**Figure 2a** **and Table S6).** However, none of them were differentially methylated in both AA case and control subjects who were exposed to CA (**Figure 2b**). In EA cases with AD, promoter regions of seven genes (*DRD5, ADH4, ALDH1A1, CHRNA5, DNMT1, OPRL1, and RGS19*) showed hypermethylation in association with CA (*P*_adjust = _0.010–0.048) (**Figure 2a** **and Table S7**). In EA control subjects, promoter regions of five genes (*GABRG1, GABRA6, ALDH1A1, OPRL1, and RGS19*) were hypermethylated in the presence of exposure to CA (*P*_adjust = _0.018–0.049) (**Figure 2a** **and Table S7).** Moreover, CA was associated with greater methylation levels in promoter regions of three genes (*ALDH1A1*, *OPRL1* and *RGS19*) in both EA case and control subjects (**Figure 2c**). When all EA samples were analyzed together, these three genes still showed hypermethylation in their promoter regions in subjects exposed to CA [*P*_adjust = _6.54×10^−4^ (*OPRL1*), 3.61×10^−3^ (*ALDH1A1*), and 1.10×10^−3^ (*RGS19*), respectively] (**Table 3**).

**Table 3:** Differentially methylated promoter regions of genes in both European American (EA) alcoholics and healthy controls who were exposed to childhood adversity (CA).

Genes	EA alcoholics (n = 136)			EA healthy controls (n = 103)			All EA subjects (n = 239)		
	PC1	PC1	*P_adj_* c	PC1	PC1	*P_adj_* c	PC1	PC1	*P_adj_* c
	(+CA)^a^	(−CA)^b^		(+CA)^a^	(−CA)^b^		(+CA)^a^	(−CA)^b^	
*OPRL1*	0.011	−0.006	0.010	0.031	−0.003	0.021	0.013	−0.005	0.0006
*ALDH1A1*	0.005	−0.002	0.025	0.020	−0.003	0.038	0.007	−0.002	0.004
*RGS19*	0.011	−0.004	0.011	0.021	−0.004	0.049	0.012	−0.004	0.001

a Principal component 1 (PC1) in subjects with childhood adversity (+CA).

b Principal component 1 (PC1) in subjects without childhood adversity (−CA).

c *P*_adj_ was calculated using multivariate linear regression analysis with adjustment of sex, age, ancestry proportion.

### Interaction of rs16969968 and CA on Methylation of CHRNA5 Promoter cg17108064

To examine whether the observed CA-associated hypermethylation of CpG cg17708064 in the promoter region of *CHRNA5* could also be affected by the functional non-synonymous variant rs16969968 in exon 5 of *CHRNA5*, we conducted a stratified analysis by rs16969968 genotypes. As shown in **Figure S2**, in EA subjects carrying genotype GG of rs16969968, multiple linear regression analysis showed that the methylation level of cg17108064 was significantly higher in subjects who experienced CA than in those who did not have such an experience (*P*_adjust_ = 0.037). Similarly, in EA subjects carrying the minor A allele of rs16969968, subjects with CA showed a significantly greater methylation level of cg17108064 than those who did not experience CA (*P*_adjust_ = 3.00×10^−4^). Further, analysis of variance showed no significant interactive effects of CA and rs16969968 genotypes on methylation levels of cg17108064, suggesting that CA might be the main source inducing greater methylation of cg17108064 in the promoter region of *CHRNA5*.

## Discussion

CA and other environmental exposures can induce epigenetic alterations, which can alter gene transcription and increase vulnerability to disease. CA-induced epigenetic changes may partially represent the molecular mechanism whereby CA predisposes to complex disorders such as AD. On the other hand, studies have shown that alcohol consumption may lead to widespread changes in DNA methylation in the genome [45]. A high prevalence of CA was noted in AD subjects included in the present study (45.9% of AA AD cases and 39.0% of EA AD cases). A high prevalence of CA in our AD subjects is consistent with the literature showing that CA is a risk factor for AD [13]. This is why we chose to examine whether CA could influence DNA methylation in AD subjects. We examined methylation levels of 384 CpGs in 82 candidate genes and observed methylation changes that were specifically associated with CA using the cross-validation approach.

We observed a population-specific association of CA with methylation alterations at certain CpG sites in specific genes. Ten CpG sites in seven genes (*ALDH1A1*, *CART*, *CHRNA5*, *HTR1B*, *OPRL1*, *PENK*, and *RGS19*) were hypermethylated in EA subjects who were exposed to CA regardless of diagnosis (**Figure 1c** and **Figure S1).** The findings were confirmed in the combined EA sample (alcoholics+healthy subjects), and *P* values of two CpG sites [cg17108064 (*CHRNA5*) and cg06031989 (*HTR1B*)] withstood Bonferroni correction. In contrast, in AAs, CA-associated CpG methylation changes could not be cross-validated in the two subgroups of samples (AD cases and healthy controls) (**Figure 1b**). The different findings for AAs and EAs might be due to (1) the distribution of adversity types in AA cases, which was significantly different from that in EA cases; (2) the distribution of numbers of adversities in AA cases, which was significantly different from that in EA cases; and (3) the distribution of comorbid substance dependence (CD, OD, MjD, or ND) in AA cases, which was significantly different from that in EA cases (refer to **Table 1**).

Because *CHRNA5* has been shown to be a susceptibility locus for multiple substance dependence disorders [33], [46], [47], [48], CA-induced methylation changes in *CHRNA5* are potentially causally related to alcohol or drug dependence. We recently showed that CA and the interaction of CA with a functional variant rs16969968 at *CHRNA5* increased the risk for nicotine dependence in EAs [14]. Bioinformatic analysis showed that CpG cg17108064 [802 bp from the transcription start site (TSS) of *CHRNA5*] is located in a potential core binding site (CCACGT) for the upstream stimulatory factor (USF, a helix-loop-helix transcription factor), consistent with the interpretation that altered methylation of cg17108064 may affect the transcription of *CHRNA5* and lead to increased risk for alcohol or drug dependence.

We analyzed whether CA-associated methylation changes in *CHRNA5* promoter CpG cg17108064 could be moderated by the *CHRNA5* non-synonymous variant rs16969968. There is evidence that methylation of certain CpG sites is correlated with single nucleotide polymorphisms (SNPs) across many cell types [49]. We examined CA-associated *CHRNA5* cg17108064 methylation changes in two genotype groups of EAs [homozygous for the major (G) allele and carriers of the minor (A) allele]. Linear regression analysis showed that *CHRNA5* cg17108064 was hypermethylated in both subgroups of EAs who had experienced CA (G/G genotype subgroup: *P*_adjust = _0.037; A/G+A/A genotype subgroup: *P*_adjust = _3.0×10^−4^). Although CA showed a higher impact on methylation of *CHRNA5* cg17108064 in A-allele carriers, no significant interactive effects of CA and genotypes of SNP rs16969968 on CpG cg17108064 methylation were observed. These findings suggest that CA could be one of the main mechanisms leading to altered methylation of *CHRNA5* promoter CpG cg17108064.

A functional study demonstrated that the minor (A) allele of *CHRNA5* rs16969968 decreased response to a nicotinic agonist [33], suggesting that the A allele was associated with lower activity of the α5 nicotinic receptor protein than the G allele. CA-induced hypermethylation of specific CpGs (such as cg17108064, which appears to be located in the core binding site of transcription factor USF) in the promoter region of *CHRNA5* may compromise *CHRNA5* transcription and produce similar clinical consequences as those produced by the rs16969968 minor (A) allele. This is consistent with the finding that hypermethylation of the promoter region decreases gene transcription [50], [51]. Although no significant interactions of CA and genotypes of *CHRNA5* rs16969968 on *CHRNA5* cg17108064 methylation were observed, we cannot exclude the possibility that CA-induced methylation changes in the *CHRNA5* promoter region and the minor (A) allele of the functional *CHRNA5* SNP rs16969968 may play a concerted role in increasing risk for alcohol or drug dependence. Both allele-specific DNA methylation (ASM) [49] and methylation quantitative trait loci (mQTL) [52] have been reported. Specifically, van IJzendoorn et al. [53] noted that interaction between methylation levels and genotypes of the serotonin transporter gene (*SLC6A4*) promoter polymorphism (5HTTLPR) predicted unresolved loss or trauma. Together, all of these findings suggest that, to understand better the mechanism of AD and other complex disorders, it is necessary to analyze the interactions of environmental factors (such as CA), epigenetic changes and DNA polymorphisms on disease susceptibility.

Additionally, we observed CA-associated overall methylation changes (by cross-validation) in promoter regions of specific genes in EAs but not in AAs. Since CA may induce methylation alterations in multiple CpG sites in a gene, we analyzed overall methylation differences in promoter regions of genes as a function of CA using principal components-based linear regression analysis. CA-associated differential methylation in promoter regions of three genes (*ALDH1A1*, *OPRL1* and *RGS19*) was cross-validated in both EA cases and controls (**Figure 2c**). Although the overall methylation level in the *CHRNA5* promoter region was altered in EA cases with CA, the finding could not be validated in EA controls with CA (**Table S7**). CA may thus have a lesser impact on methylation of seven other *CHRNA5* CpGs (8 *CHRNA5* CpGs were included in the study) in comparison to *CHRNA5* promoter CpG cg17108064, which showed differential methylation in EAs exposed to CA. However, when the sample size was enlarged by combining all EA samples together, CA-associated promoter methylation changes in *CHRNA5* were observed (**Table S7**). In AAs, CA-associated individual CpG or gene-level methylation changes in AD cases could not be validated in healthy controls. We cannot provide a good explanation for this phenomenon. Possibly, CA does not significantly influence the methylation levels of these CpGs (or genes) or those CpGs whose methylation can be altered by CA in AAs were not included in this methylation array. Additionally, some background factors differed between AAs who were selected and those AAs who were not selected for the present study. When looking into the source population comprised of 2,881 AAs and 2,380 EAs that were assessed with the SSADDA diagnostic tool, 48.4% of selected AAs while 60.0% of unselected AAs were affected with AD. The mean age of 281 selected AAs was 40±11 years, which is a little younger than that (42±9) of unselected AAs. Moreover, the proportion of males (36.6%) in selected AAs was less than that (54.4%) in unselected AAs. In contrast, no significant difference was found between selected and unselected EAs in AD proportion (selected EAs: 57.0%; unselected EAs: 52.7%), mean age (selected EAs: 39±14; unselected EAs: 40±13), and the percentage of male subjects (selected EAs: 52.3%; unselected EAs: 52.2%).

Three limitations in the present study should be noted. First, CA was retrospectively recalled by subjects. This could lead to inaccurate recall in subjects who suffered from poor mental functioning due to either the adverse events they experienced or the substances they used chronically. Second, as we know, the brain is the tissue of most interest. However, the DNA samples in this study were extracted from peripheral blood. Methylation levels of genes detected in the blood may not reflect those in the brain, either generally or in specific brain regions. The use of human postmortem brain tissue would potentially address this limitation. Third, this study investigated the effect of CA on methylation in a limited number of candidate genes. Since AD and other complex disorders are caused by multiple susceptibility genes and gene-environment interactions, a high-density genome-wide DNA methylation array study may be required to clarify the epigenetic mechanism of CA in these disorders.

Despite these limitations, this study is one of the first to investigate the association between CA and DNA methylation using a customized DNA methylation array-based assay. We found that CA may induce altered methylation of CpG sites in promoter regions of specific genes. Given the dynamic nature of epigenetic modifications, our findings suggest that changing methylation levels of specific genes may be a fruitful preventive or therapeutic approach for people exposed to CA.

## Supporting Information

Figure S1 **Box plots of methylation levels of 10 CpGs in European American (EA) alcohol dependent cases and controls with and without childhood adversity (CA).**
(DOC)

Figure S2 **Box plotting of** ***CHRNA5*** **promoter cg17108064 methylation differences between subgroups of subjects stratified by genotypes of** ***CHRNA*** **5 SNP rs16969968.**
(DOC)

Table S1 **Information of 384 CpGs in promoter regions of 82 candidate genes.**
(DOC)

Table S2 **Differentially methylated CpGs in African American (AA) alcoholics who were exposed to childhood adversity (CA).**
(DOC)

Table S3 **Differentially methylated CpGs in European American (EA) alcoholics who were exposed to childhood adversity (CA).**
(DOC)

Table S4 **Differentially methylated CpGs in African American (AA) healthy controls who were exposed to childhood adversity (CA).**
(DOC)

Table S5 **Differentially methylated CpGs in European American (EA) healthy controls who were exposed to childhood adversity (CA).**
(DOC)

Table S6 **Differentially methylated promoter regions of genes in African Americans (AAs) who were exposed to childhood adversity (CA).**
(DOC)

Table S7 **Differentially methylated promoter regions of genes in European Americans (EAs) who were exposed to childhood adversity (CA).**
(DOC)
