# Supplementary material for: Profiling of Childhood Adversity-Associated DNA Methylation Changes in Alcoholic Patients and Healthy Controls
Source: PLoS One. 2013 Jun 14;8(6):e65648. doi: 10.1371/journal.pone.0065648 (PMC3683055; doi:10.1371/journal.pone.0065648)
Supplement: Figure S1 — Box plots of methylation levels of 10 CpGs in European American (EA) alcohol dependent cases and controls with and without childhood adversity (CA). (DOC) [file pone.0065648.s001.doc]

**
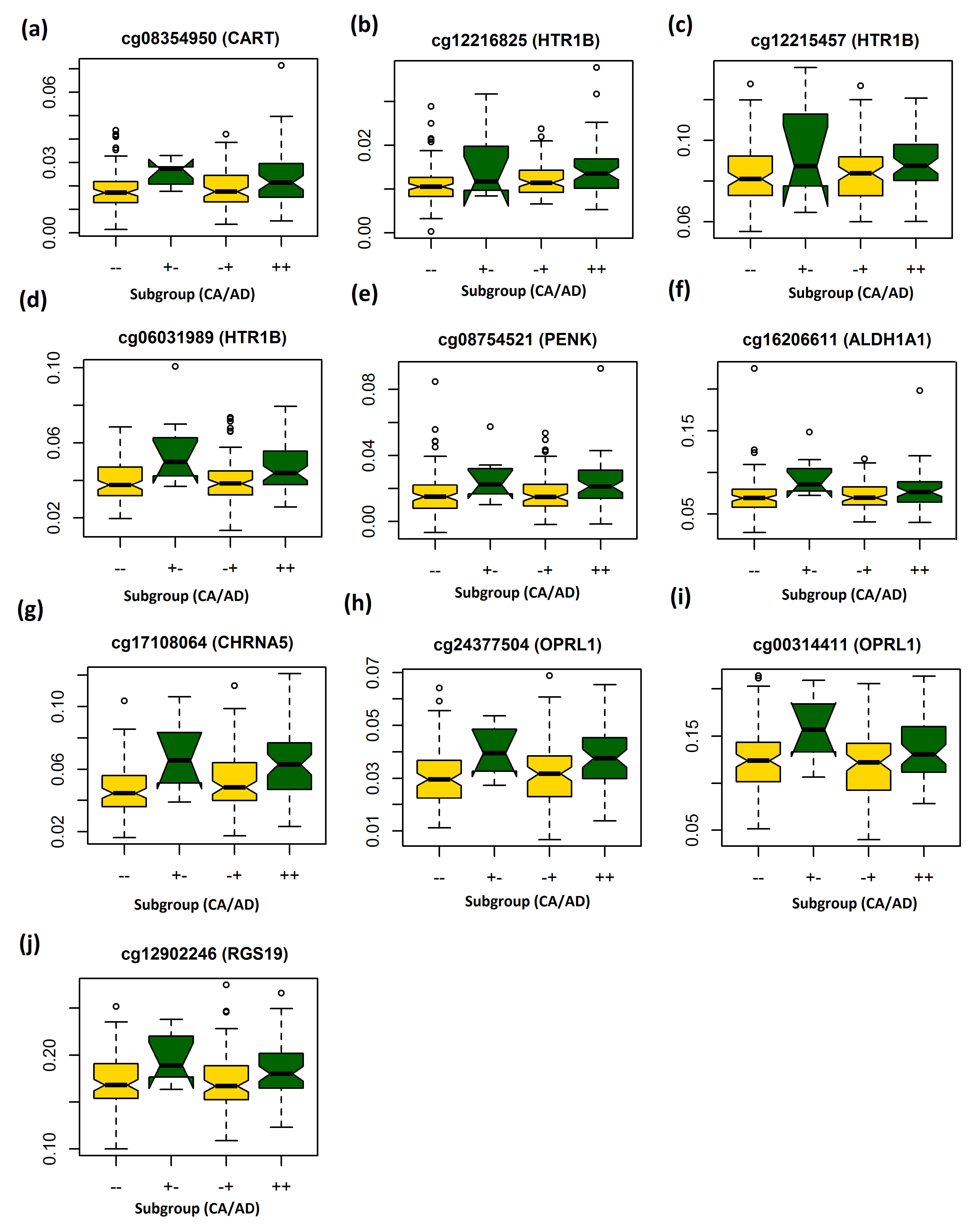
**

**Figure S1.** **Box plots of methylation levels of 10 differentially methylated CpGs in European Americans (EAs) exposed to childhood adversity (CA).**

Methylation levels of these 10 CpGs were compared between healthy subjects with CA (+/-) and without CA (-/-) as well as between alcoholics with CA (+/+) and without CA (-/+) using mulitple linear regress analysis.
