# Supplementary material for: Profiling of Childhood Adversity-Associated DNA Methylation Changes in Alcoholic Patients and Healthy Controls
Source: PLoS One. 2013 Jun 14;8(6):e65648. doi: 10.1371/journal.pone.0065648 (PMC3683055; doi:10.1371/journal.pone.0065648)
Supplement: Figure S2 — Box plotting of CHRNA5 promoter cg17108064 methylation differences between subgroups of subjects stratified by genotypes of CHRNA 5 SNP rs16969968. (DOC) [file pone.0065648.s002.doc]

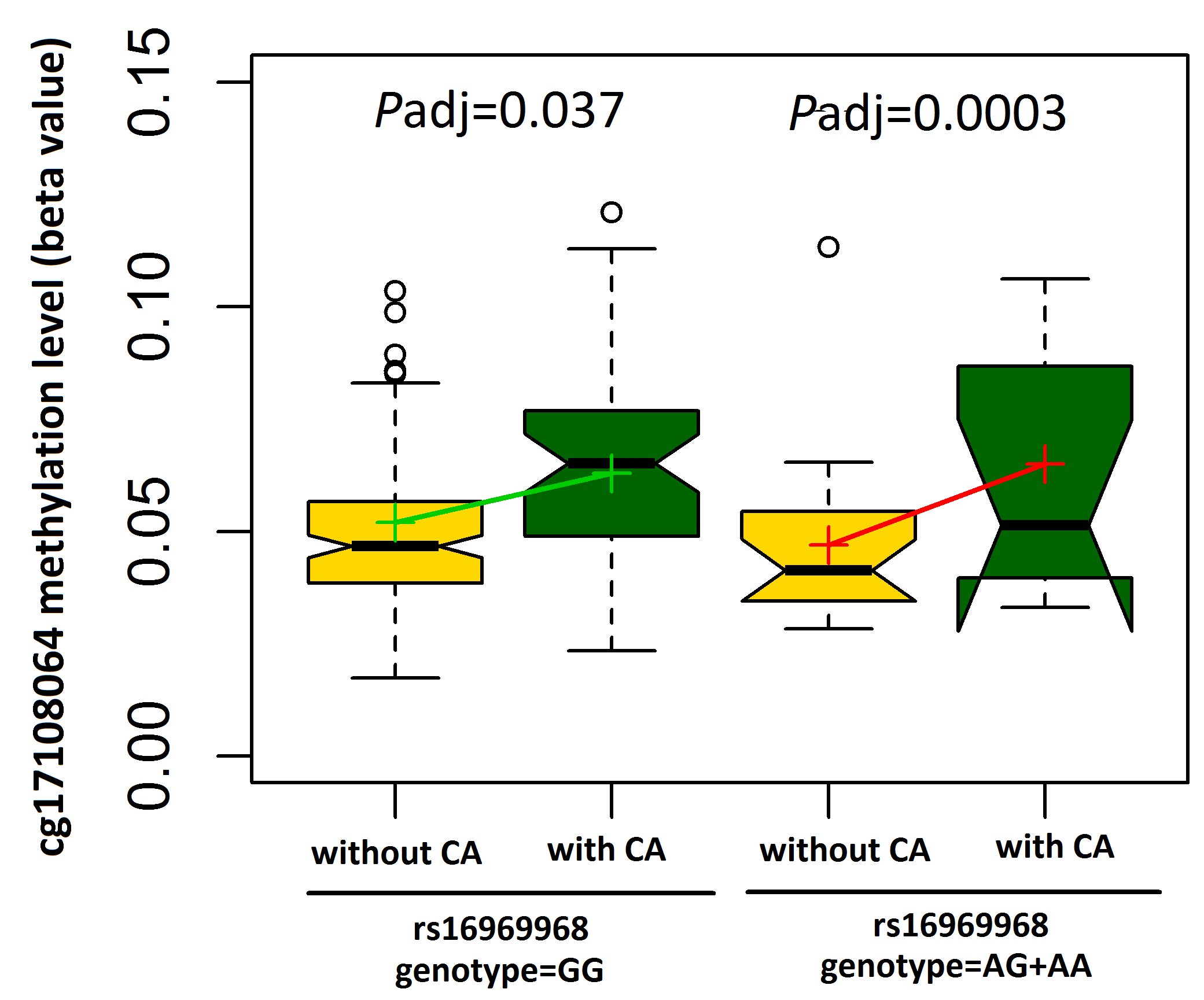


**Figure S2. Box plotting of *CHRNA5* promoter cg17108064 methylation differences between subgroups of subjects stratified by genotypes of *CHRNA5* SNP rs16969968.**
