# Supplementary material for: Profiling of Childhood Adversity-Associated DNA Methylation Changes in Alcoholic Patients and Healthy Controls
Source: PLoS One. 2013 Jun 14;8(6):e65648. doi: 10.1371/journal.pone.0065648 (PMC3683055; doi:10.1371/journal.pone.0065648)
Supplement: Table S1 — Information of 384 CpGs in promoter regions of 82 candidate genes. (DOC) [file pone.0065648.s003.doc]

**Table S1.** Information of 384 CpGs in promoter regions of 82 candidate genes.

| CpG ID | CpG ID (Illumina) | Chromosome | Coordinate ^a^ | *Gene* |
| --- | --- | --- | --- | --- |
| 1 | cg16514318 | 1 | 29009502 | *OPRD1* |
| 2 | cg06063362 | 1 | 29011014 | *OPRD1* |
| 3 | cg01706569 | 1 | 29011768 | *OPRD1* |
| 4 | cg14905768 | 1 | 29011843 | *OPRD1* |
| 5 | cg18974026 | 1 | 29012064 | *OPRD1* |
| 6 | cg01163502 | 1 | 29012185 | *OPRD1* |
| 7 | cg11487886 | 2 | 25244029 | *POMC* |
| 8 | cg06315187 | 2 | 25244419 | *POMC* |
| 9 | cg11227734 | 2 | 25244702 | *POMC* |
| 10 | cg01449287 | 2 | 25244857 | *POMC* |
| 11 | cg00293936 | 2 | 25245009 | *POMC* |
| 12 | cg02577095 | 2 | 25245228 | *POMC* |
| 13 | cg17915420 | 2 | 25246089 | *POMC* |
| 14 | cg20518314 | 2 | 25418829 | *DNMT3A* |
| 15 | cg26647484 | 2 | 25419043 | *DNMT3A* |
| 16 | cg01045241 | 2 | 25419914 | *DNMT3A* |
| 17 | cg14602957 | 2 | 25420142 | *DNMT3A* |
| 18 | cg19126369 | 2 | 171379759 | *GAD1* |
| 19 | cg22089561 | 2 | 171380171 | *GAD1* |
| 20 | cg04123893 | 2 | 171380892 | *GAD1* |
| 21 | cg17466970 | 2 | 171381124 | *GAD1* |
| 22 | cg08684483 | 2 | 171381706 | *GAD1* |
| 23 | cg19289837 | 2 | 171382135 | *GAD1* |
| 24 | cg00848742 | 2 | 208101141 | *CREB1* |
| 25 | cg17672157 | 2 | 208101551 | *CREB1* |
| 26 | cg14544856 | 2 | 208102228 | *CREB1* |
| 27 | cg14150516 | 2 | 208103029 | *CREB1* |
| 28 | cg01668758 | 2 | 208103715 | *CREB1* |
| 29 | cg03061563 | 2 | 231698652 | *HTR2B* |
| 30 | cg00618092 | 2 | 231699329 | *HTR2B* |
| 31 | cg27531267 | 2 | 231699986 | *HTR2B* |
| 32 | cg23798509 | 3 | 11008878 | *SLC6A1* |
| 33 | cg20573040 | 3 | 11009247 | *SLC6A1* |
| 34 | cg23296469 | 3 | 11009493 | *SLC6A1* |
| 35 | cg06914402 | 3 | 11009936 | *SLC6A1* |
| 36 | cg02889910 | 3 | 11010147 | *SLC6A1* |
| 37 | cg20574282 | 3 | 130641141 | *MBD4* |
| 38 | cg20346122 | 3 | 130641324 | *MBD4* |
| 39 | cg18574886 | 3 | 130641734 | *MBD4* |
| 40 | cg22851911 | 3 | 130642065 | *MBD4* |
| 41 | cg14109444 | 3 | 130642193 | *MBD4* |
| 42 | cg17494568 | 4 | 9390759 | *DRD5* |
| 43 | cg16941825 | 4 | 9392240 | *DRD5* |
| 44 | cg05931924 | 4 | 9392369 | *DRD5* |
| 45 | cg05553976 | 4 | 9392699 | *DRD5* |
| 46 | cg22515311 | 4 | 9392880 | *DRD5* |
| 47 | cg20494803 | 4 | 9392991 | *DRD5* |
| 48 | cg16772518 | 4 | 9393353 | *DRD5* |
| 49 | cg18031916 | 4 | 45820704 | *GABRG1* |
| 50 | cg23691961 | 4 | 45821130 | *GABRG1* |
| 51 | cg12139952 | 4 | 45821970 | *GABRG1* |
| 52 | cg12235279 | 4 | 45822093 | *GABRG1* |
| 53 | cg20467969 | 4 | 46086119 | *GABRA2* |
| 54 | cg02340737 | 4 | 46086402 | *GABRA2* |
| 55 | cg05807600 | 4 | 46087619 | *GABRA2* |
| 56 | cg11155145 | 4 | 46689583 | *GABRA4* |
| 57 | cg19303308 | 4 | 46689658 | *GABRA4* |
| 58 | cg01732618 | 4 | 46689934 | *GABRA4* |
| 59 | cg03593419 | 4 | 46690231 | *GABRA4* |
| 60 | cg23193606 | 4 | 46690496 | *GABRA4* |
| 61 | cg16755630 | 4 | 46728031 | *GABRB1* |
| 62 | cg02968741 | 4 | 46728180 | *GABRB1* |
| 63 | cg21074850 | 4 | 46729246 | *GABRB1* |
| 64 | cg24285775 | 4 | 100228415 | *ADH5* |
| 65 | cg17610361 | 4 | 100228846 | *ADH5* |
| 66 | cg12570007 | 4 | 100229045 | *ADH5* |
| 67 | cg18423960 | 4 | 100229673 | *ADH5* |
| 68 | cg12011299 | 4 | 100284569 | *ADH4* |
| 69 | cg26409348 | 4 | 100285646 | *ADH4* |
| 70 | cg25997474 | 4 | 100492992 | *ADH1C* |
| 71 | cg01115034 | 5 | 1497607 | *SLC6A3* |
| 72 | cg00825193 | 5 | 1497910 | *SLC6A3* |
| 73 | cg00295802 | 5 | 1498084 | *SLC6A3* |
| 74 | cg00037218 | 5 | 1498390 | *SLC6A3* |
| 75 | cg11861961 | 5 | 1498711 | *SLC6A3* |
| 76 | cg07081615 | 5 | 1498835 | *SLC6A3* |
| 77 | cg02651732 | 5 | 1499517 | *SLC6A3* |
| 78 | cg26300748 | 5 | 63292413 | *HTR1A* |
| 79 | cg13890276 | 5 | 63292581 | *HTR1A* |
| 80 | cg16740031 | 5 | 63292793 | *HTR1A* |
| 81 | cg21857413 | 5 | 63293021 | *HTR1A* |
| 82 | cg22646454 | 5 | 63293212 | *HTR1A* |
| 83 | cg00669076 | 5 | 63293383 | *HTR1A* |
| 84 | cg23759848 | 5 | 63293484 | *HTR1A* |
| 85 | cg05293338 | 5 | 63294378 | *HTR1A* |
| 86 | cg26927763 | 5 | 71050448 | *CART* |
| 87 | cg19285359 | 5 | 71050637 | *CART* |
| 88 | cg08354950 | 5 | 71051023 | *CART* |
| 89 | cg19057248 | 5 | 71051470 | *CART* |
| 90 | cg06711394 | 5 | 160907138 | *GABRB2* |
| 91 | cg02095443 | 5 | 160908531 | *GABRB2* |
| 92 | cg01677874 | 5 | 160908858 | *GABRB2* |
| 93 | cg00220369 | 5 | 161045241 | *GABRA6* |
| 94 | cg12676896 | 5 | 161045868 | *GABRA6* |
| 95 | cg24799561 | 5 | 161207199 | *GABRA1* |
| 96 | cg19518651 | 5 | 161207945 | *GABRA1* |
| 97 | cg10000484 | 5 | 161427014 | *GABRG2* |
| 98 | cg18219951 | 5 | 161427388 | *GABRG2* |
| 99 | cg01709006 | 5 | 161427678 | *GABRG2* |
| 100 | cg24642067 | 5 | 161428018 | *GABRG2* |
| 101 | cg10466664 | 5 | 161428088 | *GABRG2* |
| 102 | cg00779299 | 5 | 174802870 | *DRD1* |
| 103 | cg15130599 | 5 | 174803257 | *DRD1* |
| 104 | cg15490013 | 5 | 174803418 | *DRD1* |
| 105 | cg22921295 | 5 | 174803673 | *DRD1* |
| 106 | cg01187684 | 5 | 174803858 | *DRD1* |
| 107 | cg16769226 | 5 | 174804705 | *DRD1* |
| 108 | cg17149245 | 5 | 174805530 | *DRD1* |
| 109 | cg16493752 | 5 | 174805654 | *DRD1* |
| 110 | cg17820491 | 6 | 78228951 | *HTR1B* |
| 111 | cg15587034 | 6 | 78229082 | *HTR1B* |
| 112 | cg25304536 | 6 | 78229201 | *HTR1B* |
| 113 | cg12603785 | 6 | 78229400 | *HTR1B* |
| 114 | cg12216825 | 6 | 78229575 | *HTR1B* |
| 115 | cg23424273 | 6 | 78229946 | *HTR1B* |
| 116 | cg12215457 | 6 | 78230242 | *HTR1B* |
| 117 | cg20648561 | 6 | 78230507 | *HTR1B* |
| 118 | cg06031989 | 6 | 78230761 | *HTR1B* |
| 119 | cg07283003 | 6 | 78230890 | *HTR1B* |
| 120 | cg07506561 | 6 | 78231024 | *HTR1B* |
| 121 | cg02811260 | 6 | 78231160 | *HTR1B* |
| 122 | cg23359453 | 6 | 88910860 | *CNR1* |
| 123 | cg02395672 | 6 | 88911005 | *CNR1* |
| 124 | cg11530112 | 6 | 88911168 | *CNR1* |
| 125 | cg00331892 | 6 | 112300691 | *FYN* |
| 126 | cg12296643 | 6 | 112300988 | *FYN* |
| 127 | cg24260762 | 6 | 112301448 | *FYN* |
| 128 | cg24723845 | 6 | 112301815 | *FYN* |
| 129 | cg15269722 | 6 | 112302030 | *FYN* |
| 130 | cg17929169 | 6 | 112302215 | *FYN* |
| 131 | cg26237037 | 6 | 153493192 | *RGS17* |
| 132 | cg03461962 | 6 | 153493474 | *RGS17* |
| 133 | cg23869328 | 6 | 153493953 | *RGS17* |
| 134 | cg12505522 | 6 | 153494537 | *RGS17* |
| 135 | cg01928350 | 6 | 153494766 | *RGS17* |
| 136 | cg06649410 | 6 | 154402176 | *OPRM1* |
| 137 | cg13887561 | 6 | 154402323 | *OPRM1* |
| 138 | cg22719623 | 6 | 154402425 | *OPRM1* |
| 139 | cg04719837 | 6 | 154402578 | *OPRM1* |
| 140 | cg02489623 | 6 | 154402700 | *OPRM1* |
| 141 | cg16865650 | 6 | 154402801 | *OPRM1* |
| 142 | cg11153544 | 7 | 50595302 | *DDC* |
| 143 | cg08792950 | 7 | 50595741 | *DDC* |
| 144 | cg24972720 | 7 | 136203999 | *CHRM2* |
| 145 | cg19130396 | 7 | 136204092 | *CHRM2* |
| 146 | cg25624924 | 7 | 136204567 | *CHRM2* |
| 147 | cg19391527 | 8 | 28229651 | *PNOC* |
| 148 | cg12040841 | 8 | 28230553 | *PNOC* |
| 149 | cg27601580 | 8 | 28230707 | *PNOC* |
| 150 | cg04000318 | 8 | 28231327 | *PNOC* |
| 151 | cg13985527 | 8 | 28231544 | *PNOC* |
| 152 | cg01840162 | 8 | 54325831 | *OPRK1* |
| 153 | cg10236526 | 8 | 54325917 | *OPRK1* |
| 154 | cg09577004 | 8 | 54326093 | *OPRK1* |
| 155 | cg07344165 | 8 | 54326330 | *OPRK1* |
| 156 | cg16734072 | 8 | 54326599 | *OPRK1* |
| 157 | cg15232722 | 8 | 54327916 | *OPRK1* |
| 158 | cg05784269 | 8 | 54925936 | *RGS20* |
| 159 | cg00582628 | 8 | 54927068 | *RGS20* |
| 160 | cg10385651 | 8 | 57520878 | *PENK* |
| 161 | cg26106216 | 8 | 57521167 | *PENK* |
| 162 | cg06671711 | 8 | 57521311 | *PENK* |
| 163 | cg24645221 | 8 | 57521482 | *PENK* |
| 164 | cg27321505 | 8 | 57521713 | *PENK* |
| 165 | cg08754521 | 8 | 57522033 | *PENK* |
| 166 | cg26818805 | 8 | 57522226 | *PENK* |
| 167 | cg19473239 | 8 | 57522492 | *PENK* |
| 168 | cg16219603 | 8 | 57523140 | *PENK* |
| 169 | cg16206611 | 9 | 74756981 | *ALDH1A1* |
| 170 | cg11548648 | 9 | 74757183 | *ALDH1A1* |
| 171 | cg18095295 | 9 | 135489518 | *DBH* |
| 172 | cg10944175 | 9 | 135490020 | *DBH* |
| 173 | cg06940827 | 9 | 135490620 | *DBH* |
| 174 | cg02928015 | 9 | 135491188 | *DBH* |
| 175 | cg01006616 | 9 | 135491995 | *DBH* |
| 176 | cg09864658 | 9 | 139150707 | *GRIN1* |
| 177 | cg14727643 | 9 | 139150982 | *GRIN1* |
| 178 | cg12791151 | 9 | 139151118 | *GRIN1* |
| 179 | cg20133817 | 9 | 139151608 | *GRIN1* |
| 180 | cg24164433 | 9 | 139151849 | *GRIN1* |
| 181 | cg03109047 | 9 | 139152507 | *GRIN1* |
| 182 | cg17259718 | 9 | 139152728 | *GRIN1* |
| 183 | cg01732192 | 9 | 139153162 | *GRIN1* |
| 184 | cg23210485 | 9 | 139153405 | *GRIN1* |
| 185 | cg08754277 | 10 | 26543784 | *GAD2* |
| 186 | cg26187884 | 10 | 26544008 | *GAD2* |
| 187 | cg11500467 | 10 | 26544586 | *GAD2* |
| 188 | cg21248332 | 10 | 26545324 | *GAD2* |
| 189 | cg19713819 | 10 | 26545708 | *GAD2* |
| 190 | cg03476087 | 10 | 26546359 | *GAD2* |
| 191 | cg02567788 | 10 | 71662285 | *PPA1* |
| 192 | cg11880892 | 10 | 71662666 | *PPA1* |
| 193 | cg24774208 | 10 | 71663037 | *PPA1* |
| 194 | cg00464020 | 10 | 71663169 | *PPA1* |
| 195 | cg13752831 | 10 | 71663541 | *PPA1* |
| 196 | cg01378239 | 10 | 71664552 | *PPA1* |
| 197 | cg20038036 | 11 | 625685 | *DRD4* |
| 198 | cg25204262 | 11 | 626156 | *DRD4* |
| 199 | cg02929485 | 11 | 626452 | *DRD4* |
| 200 | cg17111401 | 11 | 626621 | *DRD4* |
| 201 | cg07385443 | 11 | 627570 | *DRD4* |
| 202 | cg12065362 | 11 | 627832 | *DRD4* |
| 203 | cg08079114 | 11 | 627981 | *DRD4* |
| 204 | cg23404860 | 11 | 628245 | *DRD4* |
| 205 | cg21272636 | 11 | 18019702 | *TPH1* |
| 206 | cg14334548 | 11 | 18769300 | *PTPN5* |
| 207 | cg13833700 | 11 | 18769927 | *PTPN5* |
| 208 | cg17484926 | 11 | 18770403 | *PTPN5* |
| 209 | cg13245417 | 11 | 18770604 | *PTPN5* |
| 210 | cg23155627 | 11 | 18770814 | *PTPN5* |
| 211 | cg17233601 | 11 | 18770994 | *PTPN5* |
| 212 | cg23079189 | 11 | 18771178 | *PTPN5* |
| 213 | cg01768936 | 11 | 18771565 | *PTPN5* |
| 214 | cg21572351 | 11 | 112335975 | *NCAM1* |
| 215 | cg14313206 | 11 | 112337308 | *NCAM1* |
| 216 | cg06777434 | 11 | 112337716 | *NCAM1* |
| 217 | cg07316621 | 11 | 112338176 | *NCAM1* |
| 218 | cg16938887 | 11 | 112690314 | *TTC12* |
| 219 | cg24462132 | 11 | 112690625 | *TTC12* |
| 220 | cg17137171 | 11 | 112691318 | *TTC12* |
| 221 | cg04915842 | 11 | 112761910 | *ANKK1* |
| 222 | cg16405454 | 11 | 112763433 | *ANKK1* |
| 223 | cg19590658 | 11 | 112763841 | *ANKK1* |
| 224 | cg11499300 | 11 | 112764262 | *ANKK1* |
| 225 | cg20203806 | 11 | 112850093 | *DRD2* |
| 226 | cg16322193 | 11 | 112850362 | *DRD2* |
| 227 | cg05421426 | 11 | 112850463 | *DRD2* |
| 228 | cg25195998 | 11 | 112850817 | *DRD2* |
| 229 | cg00243951 | 11 | 112850934 | *DRD2* |
| 230 | cg01906966 | 11 | 113279424 | *HTR3B* |
| 231 | cg08989585 | 11 | 113350239 | *HTR3A* |
| 232 | cg20621129 | 11 | 113351214 | *HTR3A* |
| 233 | cg02404574 | 12 | 14024719 | *GRIN2B* |
| 234 | cg04550775 | 12 | 14024902 | *GRIN2B* |
| 235 | cg27643501 | 12 | 14025056 | *GRIN2B* |
| 236 | cg05893218 | 12 | 14025390 | *GRIN2B* |
| 237 | cg00493111 | 12 | 14025456 | *GRIN2B* |
| 238 | cg21590372 | 12 | 14026300 | *GRIN2B* |
| 239 | cg18655110 | 12 | 110688136 | *ALDH2* |
| 240 | cg13941250 | 12 | 110688272 | *ALDH2* |
| 241 | cg21239013 | 12 | 110688555 | *ALDH2* |
| 242 | cg10959984 | 12 | 110688763 | *ALDH2* |
| 243 | cg15408490 | 12 | 110688974 | *ALDH2* |
| 244 | cg11229290 | 13 | 46367888 | *HTR2A* |
| 245 | cg15268261 | 13 | 46369029 | *HTR2A* |
| 246 | cg06020661 | 13 | 46370139 | *HTR2A* |
| 247 | cg17449649 | 15 | 24569555 | *GABRB3* |
| 248 | cg14471429 | 15 | 24569644 | *GABRB3* |
| 249 | cg17771682 | 15 | 24569866 | *GABRB3* |
| 250 | cg01690182 | 15 | 24569967 | *GABRB3* |
| 251 | cg15234319 | 15 | 24570486 | *GABRB3* |
| 252 | cg07763397 | 15 | 24571958 | *GABRB3* |
| 253 | cg02225257 | 15 | 24741680 | *GABRA5* |
| 254 | cg17525249 | 15 | 24742740 | *GABRA5* |
| 255 | cg24244000 | 15 | 25343930 | *GABRG3* |
| 256 | cg01884662 | 15 | 30109439 | *CHRNA7* |
| 257 | cg15291052 | 15 | 30109552 | *CHRNA7* |
| 258 | cg05578260 | 15 | 30109702 | *CHRNA7* |
| 259 | cg10592946 | 15 | 30110163 | *CHRNA7* |
| 260 | cg17108064 | 15 | 76644115 | *CHRNA5* |
| 261 | cg10615371 | 15 | 76644210 | *CHRNA5* |
| 262 | cg22498099 | 15 | 76644626 | *CHRNA5* |
| 263 | cg06938800 | 15 | 76644836 | *CHRNA5* |
| 264 | cg08876474 | 15 | 76645023 | *CHRNA5* |
| 265 | cg23137284 | 15 | 76645276 | *CHRNA5* |
| 266 | cg25483501 | 15 | 76645443 | *CHRNA5* |
| 267 | cg11521282 | 15 | 76645767 | *CHRNA5* |
| 268 | cg20650766 | 15 | 76699527 | *CHRNA3* |
| 269 | cg22848316 | 15 | 76700177 | *CHRNA3* |
| 270 | cg17987474 | 15 | 76700367 | *CHRNA3* |
| 271 | cg09744251 | 15 | 76700606 | *CHRNA3* |
| 272 | cg16570223 | 15 | 76701494 | *CHRNA3* |
| 273 | cg19135956 | 15 | 76719822 | *CHRNB4* |
| 274 | cg10550369 | 15 | 76720249 | *CHRNB4* |
| 275 | cg05191437 | 15 | 76720491 | *CHRNB4* |
| 276 | cg06319384 | 15 | 76720640 | *CHRNB4* |
| 277 | cg04527961 | 15 | 76720756 | *CHRNB4* |
| 278 | cg01325191 | 15 | 76721233 | *CHRNB4* |
| 279 | cg18620600 | 15 | 76721875 | *CHRNB4* |
| 280 | cg24591506 | 16 | 10183405 | *GRIN2A* |
| 281 | cg02585344 | 16 | 10183593 | *GRIN2A* |
| 282 | cg23463131 | 16 | 10183911 | *GRIN2A* |
| 283 | cg03088662 | 16 | 10184420 | *GRIN2A* |
| 284 | cg00133192 | 17 | 25586147 | *SLC6A4* |
| 285 | cg05286097 | 17 | 25586390 | *SLC6A4* |
| 286 | cg26258452 | 17 | 25586591 | *SLC6A4* |
| 287 | cg14534584 | 17 | 25587232 | *SLC6A4* |
| 288 | cg18584905 | 17 | 25587426 | *SLC6A4* |
| 289 | cg05964444 | 17 | 25588133 | *SLC6A4* |
| 290 | cg10670893 | 17 | 35036023 | *PPP1R1B* |
| 291 | cg07975378 | 17 | 35036257 | *PPP1R1B* |
| 292 | cg00112517 | 17 | 35036537 | *PPP1R1B* |
| 293 | cg08411435 | 17 | 35037550 | *PPP1R1B* |
| 294 | cg20672496 | 17 | 45581875 | *PPP1R9B* |
| 295 | cg01579505 | 17 | 45582155 | *PPP1R9B* |
| 296 | cg00161794 | 17 | 45582549 | *PPP1R9B* |
| 297 | cg15244476 | 17 | 45582714 | *PPP1R9B* |
| 298 | cg26524348 | 17 | 45583444 | *PPP1R9B* |
| 299 | cg23209660 | 17 | 45584207 | *PPP1R9B* |
| 300 | cg26566103 | 17 | 60614977 | *RGS9* |
| 301 | cg14993952 | 17 | 70367117 | *GRIN2C* |
| 302 | cg17354190 | 17 | 70367659 | *GRIN2C* |
| 303 | cg10794519 | 17 | 70367862 | *GRIN2C* |
| 304 | cg15123533 | 17 | 70368359 | *GRIN2C* |
| 305 | cg26041285 | 17 | 70368724 | *GRIN2C* |
| 306 | cg00001938 | 17 | 70369041 | *GRIN2C* |
| 307 | cg05884848 | 17 | 70369423 | *GRIN2C* |
| 308 | cg04312520 | 17 | 70369601 | *GRIN2C* |
| 309 | cg20058896 | 18 | 46061273 | *MBD1* |
| 310 | cg10390074 | 18 | 46061476 | *MBD1* |
| 311 | cg15606313 | 18 | 46062070 | *MBD1* |
| 312 | cg24863581 | 18 | 46062563 | *MBD1* |
| 313 | cg08289627 | 18 | 46062869 | *MBD1* |
| 314 | cg05788582 | 18 | 46063280 | *MBD1* |
| 315 | cg22358797 | 18 | 50004186 | *MBD2* |
| 316 | cg17388934 | 18 | 50005110 | *MBD2* |
| 317 | cg22224310 | 18 | 50005261 | *MBD2* |
| 318 | cg12511279 | 18 | 50006567 | *MBD2* |
| 319 | cg12966714 | 19 | 1542898 | *MBD3* |
| 320 | cg21372728 | 19 | 1543111 | *MBD3* |
| 321 | cg06281629 | 19 | 1544215 | *MBD3* |
| 322 | cg22531992 | 19 | 1544803 | *MBD3* |
| 323 | cg19018954 | 19 | 10166090 | *DNMT1* |
| 324 | cg08642921 | 19 | 10166280 | *DNMT1* |
| 325 | cg08339494 | 19 | 10166472 | *DNMT1* |
| 326 | cg14738521 | 19 | 10166724 | *DNMT1* |
| 327 | cg20787301 | 19 | 53588165 | *GRIN2D* |
| 328 | cg06652524 | 19 | 53588716 | *GRIN2D* |
| 329 | cg02945241 | 19 | 53588921 | *GRIN2D* |
| 330 | cg15246991 | 19 | 53589330 | *GRIN2D* |
| 331 | cg01702186 | 19 | 53589710 | *GRIN2D* |
| 332 | cg09200586 | 19 | 53589855 | *GRIN2D* |
| 333 | cg10400239 | 20 | 1922045 | *PDYN* |
| 334 | cg23617770 | 20 | 1922524 | *PDYN* |
| 335 | cg09488889 | 20 | 1923325 | *PDYN* |
| 336 | cg00164724 | 20 | 1923406 | *PDYN* |
| 337 | cg17128529 | 20 | 1923518 | *PDYN* |
| 338 | cg16164153 | 20 | 30814431 | *DNMT3B* |
| 339 | cg07170180 | 20 | 30814651 | *DNMT3B* |
| 340 | cg21235334 | 20 | 30814832 | *DNMT3B* |
| 341 | cg03490309 | 20 | 61462346 | *CHRNA4* |
| 342 | cg09897477 | 20 | 61463520 | *CHRNA4* |
| 343 | cg06365654 | 20 | 61463755 | *CHRNA4* |
| 344 | cg23806474 | 20 | 61464027 | *CHRNA4* |
| 345 | cg24172264 | 20 | 61464272 | *CHRNA4* |
| 346 | cg23593402 | 20 | 61464379 | *CHRNA4* |
| 347 | cg11829658 | 20 | 61464528 | *CHRNA4* |
| 348 | cg22872776 | 20 | 62181094 | *OPRL1* |
| 349 | cg11722562 | 20 | 62181521 | *OPRL1* |
| 350 | cg15244006 | 20 | 62181926 | *OPRL1* |
| 351 | cg16024485 | 20 | 62182186 | *OPRL1* |
| 352 | cg03896970 | 20 | 62182313 | *OPRL1* |
| 353 | cg24377504 | 20 | 62182449 | *OPRL1* |
| 354 | cg00314411 | 20 | 62182629 | *OPRL1* |
| 355 | cg12902246 | 20 | 62182990 | *RGS19* |
| 356 | cg18989937 | 20 | 62183147 | *RGS19* |
| 357 | cg20391608 | 22 | 18308581 | *COMT* |
| 358 | cg03446957 | 22 | 18309130 | *COMT* |
| 359 | cg22196019 | 22 | 18309697 | *COMT* |
| 360 | cg05989757 | 22 | 20551158 | *MAPK1* |
| 361 | cg17659879 | 22 | 20551906 | *MAPK1* |
| 362 | cg01196743 | 22 | 20552424 | *MAPK1* |
| 363 | cg22917359 | 22 | 20553228 | *MAPK1* |
| 364 | cg27580859 | X | 43398803 | *MAOA* |
| 365 | cg14009433 | X | 43399450 | *MAOA* |
| 366 | cg09356486 | X | 43400106 | *MAOA* |
| 367 | cg09883163 | X | 43400508 | *MAOA* |
| 368 | cg12338263 | X | 43626077 | *MAOB* |
| 369 | cg20550533 | X | 43626471 | *MAOB* |
| 370 | cg05876300 | X | 43626728 | *MAOB* |
| 371 | cg26000542 | X | 43626834 | *MAOB* |
| 372 | cg24323887 | X | 113722831 | *HTR2C* |
| 373 | cg17636534 | X | 113723566 | *HTR2C* |
| 374 | cg19491443 | X | 113723917 | *HTR2C* |
| 375 | cg23623863 | X | 113724321 | *HTR2C* |
| 376 | cg05010363 | X | 113724729 | *HTR2C* |
| 377 | cg02156408 | X | 113725033 | *HTR2C* |
| 378 | cg06841599 | X | 113725322 | *HTR2C* |
| 379 | cg06840457 | X | 113725764 | *HTR2C* |
| 380 | cg04055210 | X | 151370101 | *GABRA3* |
| 381 | cg04941592 | X | 153016721 | *MECP2* |
| 382 | cg11722044 | X | 153016992 | *MECP2* |
| 383 | cg05408953 | X | 153017705 | *MECP2* |
| 384 | cg05227791 | X | 153017847 | *MECP2* |

^a^ Physical position of CpG sites was annotated based on human reference sequence UCSC hg18 (NCBI build 36.1).
