# Supplementary material for: Profiling of Childhood Adversity-Associated DNA Methylation Changes in Alcoholic Patients and Healthy Controls
Source: PLoS One. 2013 Jun 14;8(6):e65648. doi: 10.1371/journal.pone.0065648 (PMC3683055; doi:10.1371/journal.pone.0065648)
Supplement: Table S2 — Differentially methylated CpGs in African American (AA) alcoholics who were exposed to childhood adversity (CA). (DOC) [file pone.0065648.s004.doc]

**Table S2.** Differentially methylated CpGs in African American (AA) alcoholics who were exposed to childhood adversity (CA) (*P_adj_* < 0.05).

| CpGs | Chr. | Position^a^ | Genes | β | |  | t-test | | FDR |  | Linear regression | |
| --- | --- | --- | --- | --- | --- | --- | --- | --- | --- | --- | --- | --- |
|  |  |  |  | +CA^b^ | -CA^c^ |  | t | *P*_nominal_^d^ | *q* |  | Effect size | *P_adj_*^e^ |
| cg07081615 | 5 | 1498835 | *SLC6A3* | 0.071 | 0.080 |  | 3.26 | 1.39E-03 | 0.534 |  | 0.01 | 1.21E-03 |
| cg20648561 | 6 | 78230507 | *HTR1B* | 0.138 | 0.120 |  | -2.56 | 1.17E-02 | 0.984 |  | -0.02 | 1.10E-02 |
| cg26237037 | 6 | 153493192 | *RGS17* | 0.027 | 0.024 |  | -2.38 | 1.86E-02 | 0.984 |  | 0.00 | 1.30E-02 |
| cg02811260 | 6 | 78231160 | *HTR1B* | 0.231 | 0.250 |  | 2.38 | 1.88E-02 | 0.984 |  | 0.02 | 1.70E-02 |
| cg26409348 | 4 | 100285646 | *ADH4* | 0.427 | 0.445 |  | 2.12 | 3.59E-02 | 0.984 |  | 0.02 | 2.74E-02 |
| cg24244000 | 15 | 25343930 | *GABRG3* | 0.435 | 0.451 |  | 2.03 | 4.48E-02 | 0.984 |  | 0.02 | 3.18E-02 |
| cg24260762 | 6 | 112301448 | *FYN* | 0.101 | 0.107 |  | 2.25 | 2.62E-02 | 0.984 |  | 0.01 | 3.22E-02 |
| cg00825193 | 5 | 1497910 | *SLC6A3* | 0.090 | 0.074 |  | -2.08 | 3.98E-02 | 0.984 |  | -0.02 | 3.77E-02 |
| cg10944175 | 9 | 135490020 | *DBH* | 0.091 | 0.084 |  | -2.13 | 3.51E-02 | 0.984 |  | -0.01 | 4.08E-02 |
| cg21857413 | 5 | 63293021 | *HTR1A* | 0.045 | 0.039 |  | -2.08 | 3.94E-02 | 0.984 |  | -0.01 | 4.57E-02 |

^a^ Physical position of CpG sites was annotated based on human reference sequence UCSC hg18 (NCBI build 36.1).

^b^ Methylation levels (β) of CpGs in subjects with childhood adversity (+CA).

^c^ Methylation levels (β) of CpGs in subjects without childhood adversity (-CA).

^d^ *P*_nominal_ was the observed *P* value calculated using empirical Bayes moderated t-test.

^e^ *P*_adj_ was the adjusted *P* value calculated using linear regression analysis with adjustment of sex, age, ancestry proportion.
