# Supplementary material for: Profiling of Childhood Adversity-Associated DNA Methylation Changes in Alcoholic Patients and Healthy Controls
Source: PLoS One. 2013 Jun 14;8(6):e65648. doi: 10.1371/journal.pone.0065648 (PMC3683055; doi:10.1371/journal.pone.0065648)
Supplement: Table S3 — Differentially methylated CpGs in European American (EA) alcoholics who were exposed to childhood adversity (CA). (DOC) [file pone.0065648.s005.doc]

**Table S3.** Differentially methylated CpGs in European American (EA) alcoholics who were exposed to childhood adversity (CA) (*P_adj_* < 0.05).

| CpGs | Chr. | Position^a^ | Genes | β | |  | t-test | | FDR |  | Linear regression | |
| --- | --- | --- | --- | --- | --- | --- | --- | --- | --- | --- | --- | --- |
|  |  |  |  | +CA^b^ | -CA^c^ |  | t | *P*_nominal_^d^ | *q* |  | Effect size | *P_adj_*^e^ |
| cg10385651 | 8 | 57520878 | *PENK* | 0.133 | 0.116 |  | 3.78 | 2.33E-04 | 0.07 |  | 0.015 | 6.17E-04 |
| cg26818805 | 8 | 57522226 | *PENK* | 0.027 | 0.020 |  | 3.61 | 4.36E-04 | 0.07 |  | 0.006 | 7.87E-04 |
| cg02404574 | 12 | 14024719 | *GRIN2B* | 0.014 | 0.012 |  | 3.12 | 2.19E-03 | 0.12 |  | 0.002 | 8.53E-04 |
| cg14905768 | 1 | 29011843 | *OPRD1* | 0.021 | 0.017 |  | 3.47 | 7.00E-04 | 0.07 |  | 0.004 | 9.05E-04 |
| cg24645221 | 8 | 57521482 | *PENK* | 0.022 | 0.017 |  | 3.40 | 8.92E-04 | 0.07 |  | 0.005 | 9.35E-04 |
| cg14334548 | 11 | 18769300 | *PTPN5* | 0.098 | 0.088 |  | 3.54 | 5.47E-04 | 0.07 |  | 0.009 | 1.54E-03 |
| cg17108064 | 15 | 76644115 | *CHRNA5* | 0.063 | 0.053 |  | 3.19 | 1.77E-03 | 0.11 |  | 0.010 | 3.15E-03 |
| cg12216825 | 6 | 78229575 | *HTR1B* | 0.015 | 0.012 |  | 2.99 | 3.27E-03 | 0.13 |  | 0.003 | 3.70E-03 |
| cg17659879 | 22 | 20551906 | *MAPK1* | 0.019 | 0.016 |  | 2.98 | 3.45E-03 | 0.13 |  | 0.002 | 5.15E-03 |
| cg06281629 | 19 | 1544215 | *MBD3* | 0.010 | 0.009 |  | 2.67 | 8.52E-03 | 0.18 |  | 0.002 | 6.80E-03 |
| cg15606313 | 18 | 46062070 | *MBD1* | 0.011 | 0.014 |  | -2.66 | 8.78E-03 | 0.18 |  | -0.003 | 6.94E-03 |
| cg08354950 | 5 | 71051023 | *CART* | 0.024 | 0.019 |  | 2.91 | 4.28E-03 | 0.15 |  | 0.005 | 7.39E-03 |
| cg06031989 | 6 | 78230761 | *HTR1B* | 0.047 | 0.040 |  | 3.00 | 3.21E-03 | 0.13 |  | 0.006 | 8.11E-03 |
| cg12215457 | 6 | 78230242 | *HTR1B* | 0.090 | 0.084 |  | 2.74 | 6.96E-03 | 0.18 |  | 0.006 | 9.22E-03 |
| cg10236526 | 8 | 54325917 | *OPRK1* | 0.035 | 0.024 |  | 2.72 | 7.37E-03 | 0.18 |  | 0.010 | 9.28E-03 |
| cg26041285 | 17 | 70368724 | *GRIN2C* | 0.052 | 0.039 |  | 2.52 | 1.28E-02 | 0.23 |  | 0.013 | 1.12E-02 |
| cg00314411 | 20 | 62182629 | *OPRL1* | 0.137 | 0.121 |  | 2.69 | 8.14E-03 | 0.18 |  | 0.015 | 1.16E-02 |
| cg22719623 | 6 | 154402425 | *OPRM1* | 0.082 | 0.075 |  | 2.72 | 7.45E-03 | 0.18 |  | 0.006 | 1.67E-02 |
| cg15130599 | 5 | 174803257 | *DRD1* | 0.224 | 0.212 |  | 2.72 | 7.37E-03 | 0.18 |  | 0.010 | 1.74E-02 |
| cg18989937 | 20 | 62183147 | *RGS19* | 0.244 | 0.236 |  | 2.47 | 1.49E-02 | 0.26 |  | 0.007 | 1.93E-02 |
| cg12902246 | 20 | 62182990 | *RGS19* | 0.185 | 0.172 |  | 2.43 | 1.63E-02 | 0.27 |  | 0.012 | 2.00E-02 |
| cg26258452 | 17 | 25586591 | *SLC6A4* | 0.199 | 0.189 |  | 2.68 | 8.34E-03 | 0.18 |  | 0.008 | 2.06E-02 |
| cg15587034 | 6 | 78229082 | *HTR1B* | 0.011 | 0.009 |  | 2.22 | 2.78E-02 | 0.34 |  | 0.001 | 2.77E-02 |
| cg08754521 | 8 | 57522033 | *PENK* | 0.023 | 0.017 |  | 2.42 | 1.70E-02 | 0.27 |  | 0.005 | 2.81E-02 |
| cg08339494 | 19 | 10166472 | *DNMT1* | 0.014 | 0.007 |  | 2.55 | 1.19E-02 | 0.23 |  | 0.006 | 2.82E-02 |
| cg16206611 | 9 | 74756981 | *ALDH1A1* | 0.079 | 0.072 |  | 2.08 | 3.96E-02 | 0.38 |  | 0.008 | 3.06E-02 |
| cg20494803 | 4 | 9392991 | *DRD5* | 0.387 | 0.352 |  | 2.30 | 2.29E-02 | 0.32 |  | 0.034 | 3.08E-02 |
| cg13833700 | 11 | 18769927 | *PTPN5* | 0.011 | 0.009 |  | 2.22 | 2.80E-02 | 0.34 |  | 0.002 | 3.09E-02 |
| cg23193606 | 4 | 46690496 | *GABRA4* | 0.011 | 0.009 |  | 2.27 | 2.49E-02 | 0.33 |  | 0.001 | 3.29E-02 |
| cg25624924 | 7 | 136204567 | *CHRM2* | 0.024 | 0.021 |  | 2.20 | 2.97E-02 | 0.35 |  | 0.002 | 3.75E-02 |
| cg24377504 | 20 | 62182449 | *OPRL1* | 0.038 | 0.033 |  | 2.40 | 1.76E-02 | 0.27 |  | 0.004 | 4.15E-02 |
| cg00669076 | 5 | 63293383 | *HTR1A* | 0.055 | 0.049 |  | 2.29 | 2.35E-02 | 0.32 |  | 0.004 | 4.77E-02 |
| cg12011299 | 4 | 100284569 | *ADH4* | 0.207 | 0.167 |  | 1.91 | 5.77E-02 | 0.43 |  | 0.042 | 4.87E-02 |
| cg03896970 | 20 | 62182313 | *OPRL1* | 0.036 | 0.033 |  | 2.09 | 3.86E-02 | 0.38 |  | 0.003 | 4.94E-02 |

^a^ Physical position of CpG sites was annotated based on human reference sequence UCSC hg18 (NCBI build 36.1).

^b^ Methylation levels (β) of CpGs in subjects with childhood adversity (+CA).

^c^ Methylation levels (β) of CpGs in subjects without childhood adversity (-CA).

^d^ *P*_nominal_ was the observed *P* value calculated using empirical Bayes moderated t-test.

^e^ *P*_adj_ was the adjusted *P* value calculated using linear regression analysis with adjustment of sex, age, ancestry proportion.
