# Supplementary material for: Profiling of Childhood Adversity-Associated DNA Methylation Changes in Alcoholic Patients and Healthy Controls
Source: PLoS One. 2013 Jun 14;8(6):e65648. doi: 10.1371/journal.pone.0065648 (PMC3683055; doi:10.1371/journal.pone.0065648)
Supplement: Table S4 — Differentially methylated CpGs in African American (AA) healthy controls who were exposed to childhood adversity (CA). (DOC) [file pone.0065648.s006.doc]

**Table S4.** Differentially methylated CpGs in African American (AA) healthy controls who were exposed to childhood adversity (CA) (*P_adj_* < 0.05).

| CpGs | Chr. | Position^a^ | Genes | β | |  | t-test | | FDR |  | Linear regression | |
| --- | --- | --- | --- | --- | --- | --- | --- | --- | --- | --- | --- | --- |
|  |  |  |  | +CA^b^ | -CA^c^ |  | t | *P*_nominal_^d^ | *q* |  | Effect size | *P_adj_*^e^ |
| cg19018954 | 19 | 10166090 | *DNMT1* | 0.019 | 0.024 |  | -2.38 | 1.86E-02 | 0.84 |  | -0.005 | 9.93E-03 |
| cg22646454 | 5 | 63293212 | *HTR1A* | 0.029 | 0.034 |  | -2.02 | 4.48E-02 | 0.84 |  | -0.005 | 1.55E-02 |
| cg14534584 | 17 | 25587232 | *SLC6A4* | 0.053 | 0.059 |  | -2.04 | 4.29E-02 | 0.84 |  | -0.007 | 1.80E-02 |
| cg03461962 | 6 | 153493474 | *RGS17* | 0.013 | 0.016 |  | -2.00 | 4.71E-02 | 0.84 |  | -0.003 | 1.98E-02 |
| cg13941250 | 12 | 110688272 | *ALDH2* | 0.944 | 0.937 |  | 2.27 | 2.48E-02 | 0.84 |  | 0.007 | 2.63E-02 |
| cg01709006 | 5 | 161427678 | *GABRG2* | 0.061 | 0.066 |  | -1.21 | 2.30E-01 | 0.86 |  | -0.007 | 2.71E-02 |
| cg11548648 | 9 | 74757183 | *ALDH1A1* | 0.055 | 0.048 |  | 1.97 | 5.03E-02 | 0.84 |  | 0.008 | 2.75E-02 |
| cg16941825 | 4 | 9392240 | *DRD5* | 0.033 | 0.037 |  | -1.89 | 6.12E-02 | 0.84 |  | -0.005 | 2.90E-02 |
| cg17771682 | 15 | 24569866 | *GABRB3* | 0.015 | 0.019 |  | -1.98 | 4.91E-02 | 0.84 |  | -0.004 | 3.11E-02 |
| cg10670893 | 17 | 35036023 | *PPP1R1B* | 0.252 | 0.228 |  | 2.08 | 3.92E-02 | 0.84 |  | 0.026 | 3.30E-02 |
| cg22089561 | 2 | 171380171 | *GAD1* | 0.057 | 0.062 |  | -2.00 | 4.74E-02 | 0.84 |  | -0.005 | 3.38E-02 |
| cg12966714 | 19 | 1542898 | *MBD3* | 0.028 | 0.033 |  | -1.98 | 5.01E-02 | 0.84 |  | -0.005 | 3.61E-02 |
| cg00164724 | 20 | 1923406 | *PDYN* | 0.528 | 0.505 |  | 2.22 | 2.79E-02 | 0.84 |  | 0.022 | 3.82E-02 |
| cg07316621 | 11 | 112338176 | *NCAM1* | 0.011 | 0.013 |  | -1.82 | 7.03E-02 | 0.84 |  | -0.002 | 3.98E-02 |
| cg00001938 | 17 | 70369041 | *GRIN2C* | 0.027 | 0.032 |  | -1.81 | 7.19E-02 | 0.84 |  | -0.006 | 4.05E-02 |
| cg05964444 | 17 | 25588133 | *SLC6A4* | 0.064 | 0.070 |  | -1.75 | 8.27E-02 | 0.84 |  | -0.007 | 4.39E-02 |
| cg18423960 | 4 | 100229673 | *ADH5* | 0.553 | 0.534 |  | 2.10 | 3.77E-02 | 0.84 |  | 0.019 | 4.49E-02 |
| cg15291052 | 15 | 30109552 | *CHRNA7* | 0.034 | 0.037 |  | -1.86 | 6.47E-02 | 0.84 |  | -0.004 | 4.62E-02 |
| cg11530112 | 6 | 88911168 | *CNR1* | 0.692 | 0.651 |  | 1.76 | 7.97E-02 | 0.84 |  | 0.047 | 4.64E-02 |

^a^ Physical position of CpG sites was annotated based on human reference sequence UCSC hg18 (NCBI build 36.1).

^b^ Methylation levels (β) of CpGs in subjects with childhood adversity (+CA).

^c^ Methylation levels (β) of CpGs in subjects without childhood adversity (-CA).

^d^ *P*_nominal_ was the observed *P* value calculated using empirical Bayes moderated t-test.

^e^ *P*_adj_ was the adjusted *P* value calculated using linear regression analysis with adjustment of sex, age, ancestry proportion.
