# Supplementary material for: Profiling of Childhood Adversity-Associated DNA Methylation Changes in Alcoholic Patients and Healthy Controls
Source: PLoS One. 2013 Jun 14;8(6):e65648. doi: 10.1371/journal.pone.0065648 (PMC3683055; doi:10.1371/journal.pone.0065648)
Supplement: Table S5 — Differentially methylated CpGs in European American (EA) healthy controls who were exposed to childhood adversity (CA). (DOC) [file pone.0065648.s007.doc]

**Table S5.** Differentially methylated CpGs in European American (EA) healthy controls who were exposed to childhood adversity (CA) (*P_adj_* < 0.05).

| CpGs | Chr. | Position^a^ | Genes | β | |  | t-test | | FDR |  | Linear regression | |
| --- | --- | --- | --- | --- | --- | --- | --- | --- | --- | --- | --- | --- |
|  |  |  |  | +CA^b^ | -CA^c^ |  | t | *P*_nominal_^d^ | *q* |  | Effect size | *P_adj_*^e^ |
| cg06031989 | 6 | 78230761 | *HTR1B* | 0.056 | 0.040 |  | 3.66 | 3.96E-04 | 0.10 |  | 0.015 | 5.28E-04 |
| cg17108064 | 15 | 76644115 | *CHRNA5* | 0.068 | 0.047 |  | 3.59 | 5.02E-04 | 0.10 |  | 0.020 | 1.25E-03 |
| cg15490013 | 5 | 174803418 | *DRD1* | 0.044 | 0.034 |  | 2.72 | 7.76E-03 | 0.51 |  | 0.010 | 5.59E-03 |
| cg21857413 | 5 | 63293021 | *HTR1A* | 0.052 | 0.038 |  | 2.63 | 9.89E-03 | 0.51 |  | 0.014 | 7.19E-03 |
| cg26409348 | 4 | 100285646 | *ADH4* | 0.411 | 0.449 |  | -2.74 | 7.31E-03 | 0.51 |  | -0.038 | 9.58E-03 |
| cg06365654 | 20 | 61463755 | *CHRNA4* | 0.024 | 0.016 |  | 2.78 | 6.54E-03 | 0.51 |  | 0.007 | 1.22E-02 |
| cg16206611 | 9 | 74756981 | *ALDH1A1* | 0.094 | 0.071 |  | 2.60 | 1.06E-02 | 0.51 |  | 0.022 | 1.46E-02 |
| cg18031916 | 4 | 45820704 | *GABRG1* | 0.312 | 0.372 |  | -1.92 | 5.82E-02 | 0.57 |  | -0.074 | 1.86E-02 |
| cg12216825 | 6 | 78229575 | *HTR1B* | 0.015 | 0.011 |  | 2.34 | 2.11E-02 | 0.57 |  | 0.004 | 2.14E-02 |
| cg00464020 | 10 | 71663169 | *PPA1* | 0.007 | 0.002 |  | 2.32 | 2.26E-02 | 0.57 |  | 0.005 | 2.19E-02 |
| cg24377504 | 20 | 62182449 | *OPRL1* | 0.040 | 0.031 |  | 2.27 | 2.51E-02 | 0.57 |  | 0.010 | 2.22E-02 |
| cg08754521 | 8 | 57522033 | *PENK* | 0.026 | 0.017 |  | 1.83 | 6.97E-02 | 0.62 |  | 0.011 | 2.23E-02 |
| cg15246991 | 19 | 53589330 | *GRIN2D* | 0.047 | 0.034 |  | 2.37 | 1.94E-02 | 0.57 |  | 0.012 | 2.39E-02 |
| cg05876300 | 23 | 43626728 | *MAOB* | 0.032 | 0.021 |  | 2.28 | 2.47E-02 | 0.57 |  | 0.009 | 2.40E-02 |
| cg22646454 | 5 | 63293212 | *HTR1A* | 0.033 | 0.025 |  | 2.49 | 1.45E-02 | 0.56 |  | 0.007 | 2.48E-02 |
| cg00314411 | 20 | 62182629 | *OPRL1* | 0.158 | 0.126 |  | 2.68 | 8.63E-03 | 0.51 |  | 0.027 | 2.63E-02 |
| cg24972720 | 7 | 136203999 | *CHRM2* | 0.035 | 0.026 |  | 2.45 | 1.61E-02 | 0.56 |  | 0.009 | 2.65E-02 |
| cg13887561 | 6 | 154402323 | *OPRM1* | 0.033 | 0.026 |  | 2.31 | 2.30E-02 | 0.57 |  | 0.007 | 3.12E-02 |
| cg11861961 | 5 | 1498711 | *SLC6A3* | 0.030 | 0.024 |  | 1.98 | 5.05E-02 | 0.57 |  | 0.007 | 3.64E-02 |
| cg00220369 | 5 | 161045241 | *GABRA6* | 0.513 | 0.545 |  | -2.04 | 4.38E-02 | 0.57 |  | -0.034 | 3.68E-02 |
| cg12902246 | 20 | 62182990 | *RGS19* | 0.197 | 0.171 |  | 2.53 | 1.29E-02 | 0.55 |  | 0.021 | 3.73E-02 |
| cg08354950 | 5 | 71051023 | *CART* | 0.025 | 0.019 |  | 2.14 | 3.46E-02 | 0.57 |  | 0.007 | 4.24E-02 |
| cg16941825 | 4 | 9392240 | *DRD5* | 0.035 | 0.027 |  | 2.21 | 2.93E-02 | 0.57 |  | 0.008 | 4.53E-02 |
| cg12235279 | 4 | 45822093 | *GABRG1* | 0.672 | 0.707 |  | -1.72 | 8.87E-02 | 0.65 |  | -0.043 | 4.58E-02 |
| cg12215457 | 6 | 78230242 | *HTR1B* | 0.094 | 0.082 |  | 2.22 | 2.84E-02 | 0.57 |  | 0.011 | 4.64E-02 |
| cg14738521 | 19 | 10166724 | *DNMT1* | 0.014 | 0.011 |  | 1.92 | 5.81E-02 | 0.57 |  | 0.004 | 4.85E-02 |

^a^ Physical position of CpG sites was annotated based on human reference sequence UCSC hg18 (NCBI build 36.1).

^b^ Methylation levels (β) of CpGs in subjects with childhood adversity (+CA).

^c^ Methylation levels (β) of CpGs in subjects without childhood adversity (-CA).

^d^ *P*_nominal_ was the observed *P* value calculated using empirical Bayes moderated t-test.

^e^ *P*_adj_ was the adjusted *P* value calculated using linear regression analysis with adjustment of sex, age, ancestry proportion.
