# Supplementary material for: Profiling of Childhood Adversity-Associated DNA Methylation Changes in Alcoholic Patients and Healthy Controls
Source: PLoS One. 2013 Jun 14;8(6):e65648. doi: 10.1371/journal.pone.0065648 (PMC3683055; doi:10.1371/journal.pone.0065648)
Supplement: Table S6 — Differentially methylated promoter regions of genes in African Americans (AAs) who were exposed to childhood adversity (CA). (DOC) [file pone.0065648.s008.doc]

**Table S6.** Differentially methylated promoter regions of genes in African Americans (AAs) who were exposed to childhood adversity (CA).

| Genes | AA alcoholics (n=135) | | |  | AA healthy controls (n=144) | | |  | All AA subjects (n=279) | | |
| --- | --- | --- | --- | --- | --- | --- | --- | --- | --- | --- | --- |
|  | PC1 | PC1 | *P_adj_* |  | PC1 | PC1 | *P_adj_*^c^ |  | PC1 | PC1 | *P_adj_*^c^ |
|  | +CA^a^ | -CA^b^ |  |  | +CA^a^ | -CA^b^ |  |  | +CA^a^ | -CA^b^ |  |
| *MAOB* | -0.027 | 0.001 | 8.81E-02 |  | -0.004 | 0.014 | 3.57E-02 |  | -0.020 | 0.009 | 7.10E-03 |
| *GABRG3* | 0.004 | -0.012 | 3.18E-02 |  | 0.007 | 0.004 | 6.67E-01 |  | 0.004 | -0.002 | 4.71E-02 |
| *GRIN2A* | -0.003 | 0.000 | 2.37E-01 |  | -0.003 | 0.002 | 4.67E-02 |  | -0.003 | 0.001 | 2.28E-02 |
| *SLC6A3* | -0.002 | 0.013 | 7.01E-02 |  | -0.008 | -0.005 | 3.10E-01 |  | -0.004 | 0.002 | 4.98E-02 |
| *ADH5* | -0.002 | -0.001 | 9.32E-01 |  | 0.017 | -0.002 | 4.74E-02 |  | 0.003 | -0.002 | 2.78E-01 |

^a^ The principal component 1 (PC1) was obtained by principal components analyses for AAs with childhood adversity (+CA).

^b^ The principal component 1 (PC1) was obtained by principal components analyses for AAs without childhood adversity (-CA).

c *P*_adj_ was calculated using linear regression analysis with adjustment of sex, age, ancestry proportion.
