# Supplementary material for: Profiling of Childhood Adversity-Associated DNA Methylation Changes in Alcoholic Patients and Healthy Controls
Source: PLoS One. 2013 Jun 14;8(6):e65648. doi: 10.1371/journal.pone.0065648 (PMC3683055; doi:10.1371/journal.pone.0065648)
Supplement: Table S7 — Differentially methylated promoter regions of genes in European Americans (EAs) who were exposed to childhood adversity (CA). (DOC) [file pone.0065648.s009.doc]

**Table S7.** Differentially methylated promoter regions of genes in European Americans (EAs) exposed to childhood adversity (CA).

| Genes | EA alcoholics (n=136) | | |  | EA healthy controls (n=103) | | |  | All EA subjects (n=239) | | |
| --- | --- | --- | --- | --- | --- | --- | --- | --- | --- | --- | --- |
|  | PC1 | PC1 | *P_adj_* |  | PC1 | PC1 | *P_adj_*^c^ |  | PC1 | PC1 | *P_adj_*^c^ |
|  | +CA^a^ | -CA^b^ |  |  | +CA^a^ | -CA^b^ |  |  | +CA^a^ | -CA^b^ |  |
| *OPRL1* | 0.011 | -0.006 | 9.77E-03 |  | 0.031 | -0.003 | 2.13E-02 |  | 0.013 | -0.005 | 6.54E-04 |
| *RGS19* | 0.011 | -0.004 | 1.07E-02 |  | 0.021 | -0.004 | 4.96E-02 |  | 0.012 | -0.004 | 1.10E-03 |
| *ALDH1A1* | 0.005 | -0.002 | 2.51E-02 |  | 0.020 | -0.003 | 3.75E-02 |  | 0.007 | -0.002 | 3.61E-03 |
| *DNMT1* | 0.003 | -0.004 | 2.31E-02 |  | 0.011 | 0.000 | 8.01E-02 |  | 0.004 | -0.001 | 7.84E-03 |
| *CHRNA5* | -0.015 | 0.000 | 3.23E-02 |  | -0.011 | 0.009 | 1.53E-01 |  | -0.014 | 0.005 | 8.69E-03 |
| *GABRA2* | 0.005 | 0.000 | 8.05E-02 |  | 0.007 | -0.003 | 1.09E-01 |  | 0.005 | -0.002 | 1.23E-02 |
| *DRD5* | 0.026 | -0.010 | 3.08E-02 |  | 0.024 | -0.007 | 2.35E-01 |  | 0.025 | -0.009 | 1.76E-02 |
| *DNMT3B* | 0.007 | -0.006 | 5.02E-02 |  | 0.011 | 0.001 | 3.69E-01 |  | 0.007 | -0.002 | 2.51E-02 |
| *GABRA1* | 0.002 | 0.000 | 5.65E-02 |  | 0.001 | -0.001 | 3.65E-01 |  | 0.002 | -0.001 | 2.62E-02 |
| *SLC6A3* | -0.019 | 0.001 | 6.07E-02 |  | -0.001 | 0.010 | 3.50E-01 |  | -0.017 | 0.006 | 2.86E-02 |
| *ALDH2* | -0.008 | 0.002 | 1.58E-01 |  | -0.027 | 0.005 | 6.56E-02 |  | -0.011 | 0.004 | 3.25E-02 |
| *GABRG2* | -0.006 | 0.002 | 1.07E-01 |  | -0.005 | 0.002 | 3.17E-01 |  | -0.006 | 0.002 | 3.42E-02 |
| *DRD2* | -0.008 | 0.000 | 6.07E-02 |  | -0.002 | 0.005 | 4.71E-01 |  | -0.007 | 0.002 | 3.71E-02 |
| *ADH4* | -0.022 | 0.018 | 4.81E-02 |  | 0.050 | -0.008 | 1.05E-01 |  | -0.012 | 0.004 | 2.32E-01 |
| *GABRA6* | 0.004 | 0.002 | 9.58E-01 |  | 0.032 | -0.006 | 4.46E-02 |  | 0.008 | -0.003 | 2.55E-01 |
| *GABRG1* | 0.017 | 0.008 | 6.47E-01 |  | -0.094 | -0.008 | 1.76E-02 |  | 0.003 | -0.001 | 6.50E-01 |

^a^ The principal component 1 (PC1) was obtained by principal components analyses for EAs with childhood adversity (+CA).

^b^ The principal component 1 (PC1) was obtained by principal components analyses for EAs without childhood adversity (-CA).

c *P*_adj_ was calculated using linear regression analysis with adjustment of sex, age, ancestry proportion.
